# Characterization of a Basidiomycota hydrophobin reveals the structural basis for a high-similarity Class I subdivision

**DOI:** 10.1038/srep45863

**Published:** 2017-04-10

**Authors:** Julie-Anne Gandier, David N. Langelaan, Amy Won, Kylie O’Donnell, Julie L. Grondin, Holly L. Spencer, Philip Wong, Elisabeth Tillier, Christopher Yip, Steven P. Smith, Emma R. Master

**Affiliations:** 1 Department of Chemical Engineering and Applied Chemistry, University of Toronto, Toronto, Ontario, Canada; 2 Department of Biochemistry & Molecular Biology, Dalhousie University, Halifax, Nova Scotia, Canada; 3 Department of Medical Biophysics, University of Toronto, Toronto, Ontario, Canada; 4 Department of Biomedical and Molecular Sciences, Queen’s University, Kingston, Ontario, Canada; 5 e-Institute of Biomaterials and Biomedical Engineering, University of Toronto, Toronto, Ontario, Canada

## Abstract

Class I hydrophobins are functional amyloids secreted by fungi. They self-assemble into organized films at interfaces producing structures that include cellular adhesion points and hydrophobic coatings. Here, we present the first structure and solution properties of a unique Class I protein sequence of Basidiomycota origin: the *Schizophyllum commune* hydrophobin SC16 (*hyd1*). While the core β-barrel structure and disulphide bridging characteristic of the hydrophobin family are conserved, its surface properties and secondary structure elements are reminiscent of both Class I and II hydrophobins. Sequence analyses of hydrophobins from 215 fungal species suggest this structure is largely applicable to a high-identity Basidiomycota Class I subdivision (IB). To validate this prediction, structural analysis of a comparatively distinct Class IB sequence from a different fungal order, namely the *Phanerochaete carnosa* PcaHyd1, indicates secondary structure properties similar to that of SC16. Together, these results form an experimental basis for a high-identity Class I subdivision and contribute to our understanding of functional amyloid formation.

Mechanisms of protein self-assembly play essential roles in cell development and survival, and offer unique biotechnological opportunities. One such family of self-assembling proteins are the hydrophobins, members of which tailor interfaces to filamentous fungal life. Hydrophobins can form functional amyloids that cover and protect spores^1^, assemble to coat fruiting bodies to repel water^2^, reduce the surface tension of liquid media to facilitate the emergence of aerial hyphae^3^, and create adhesion points on surfaces^3^. While hydrophobins have been used for foam stabilization in industrial settings^4^, the range of potential applications for this protein family (reviewed in refs 5, 6, 7) is as wide as the spectrum of properties it displays in Nature.

Hydrophobins are low molecular weight (5–20 kDa) proteins that can be identified by a conserved pattern of eight cysteine residues that form four disulphide bonds^8^. Conventionally, hydrophobins have been divided into two classes based on spacing of the cysteine residues, position of hydrophobic residues relative to these cysteines, and solution properties of their assemblies^9^. Class I hydrophobins are produced by ascomycota and basidiomycota fungi, and form assemblies of amyloid-like rodlets that are SDS insoluble, resistant to heat, and resistant to acidic conditions^10^. Class II hydrophobins, which to date have only been identified among the ascomycota, are readily identified as their sequences are comparatively conserved. Their assemblies are less stable than Class I assemblies and can be disassociated by SDS-alcohol mixtures^10^. Common methods to identify a hydrophobin as Class I include detection of rodlet-shaped assemblies by electron microscopy^11^ or atomic force microscopy^12^, and binding assays using amyloid specific dyes, such as Thioflavin T^12^ or Congo red^13^.

Recent genomic initiatives to characterize fungi have dramatically increased the number of predicted hydrophobin sequences. While putative Class II hydrophobins are still readily identified by sequence analysis, it remains difficult to reach a single consensus as to a Class I signature, leading to the proposition for further subdivision of the class^14^,^15^,^16^. Considering phylogenetic analyses of sequences from a wide range of fungi, bootstrap values support a separation of Class I ascomycota and basidiomycota sequences but are too low to confidently support further subdivisions^17^,^18^. It is also important to note that while the number of predicted sequences increases, both the forces driving hydrophobin gene evolution and the roles of individual genes remain largely unclear^18^.

While hydrophobins from both phyla have been characterized, high-resolution structures exist only for those of ascomycota origin: the Class I EAS, DewA, MPG1, and Class II NC2, HFBI, and HFBII^8^,^19^,^20^,^21^,^22^,^23^. To address whether basidiomycota hydrophobins share structural and functional features with these proteins, we preliminarily characterized SC16 (*hyd1*) from *Schizophyllum commune* and determined its structure. SC16 adopts a distinct structural topology reminiscent of both Class I and Class II hydrophobins. Bioinformatics analyses of predicted hydrophobin protein sequences from 215 fungal species reveal that SC16 is representative of a high identity basidiomycota subdivision of Class I (Class IB). This finding was supported by the NMR-based analysis of the Class IB *P. carnosa* hydrophobin PcaHyd1.

## Results and Discussion

### SC16 demonstrates Class I properties

The *S. commune* hydrophobin SC3 is one of the best functionally characterized basidiomycota Class I hydrophobins to date. However, its ability to self-assemble at 100 μg/ml without significant aeration^12^ precluded its detailed structural characterization. Sequence analysis of the *S. commune* hydrophobins identified SC16 as sharing 56% sequence identity with SC3 from its first to last cysteine residues (Figure S1) and preliminary NMR studies confirmed the presence of SC16 monomers in solution at protein concentrations required for structural studies. As such, we hypothesized that while the assembly kinetics of SC16 differed from those of SC3, given their sequence identity, SC16 would reveal functional properties largely consistent with SC3, while offering properties amenable to our characterization approach.

**Figure 1:**
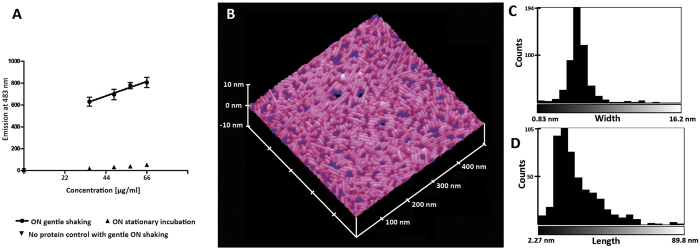
SC16 is an active Class I hydrophobin. (**A**) Linear dependency of enhanced fluorescence of Thioflavin T induced by aeration and protein concentration. Gentle head over tail overnight aeration on a rotary shaker induced enhanced ThT fluorescence. N = 5 for experiment while N = 3 for control (no shaking and no protein). All error bars indicate standard deviation. (**B**) AFM image of dried down SC16 in water (aerated) on HOPG reveals SC16 assembled into rodlets. Model prepared using Nanoscope software (version 5.12r3) (**C**) Width, and (**D**) length distributions indicates SC16 elongates after initial assembly. The average thickness of the layer is 1.99 ± 0.82 nm.

The solution properties of recombinant SC16 produced in *Escherichia coli* were consistent with those of other Class I hydrophobins^10^. Specifically, upon introduction of an air-water interface by gentle head over tail shaking (i.e. aeration) specific binding to the amyloid specific dye ThT was induced as indicated by the linear relationship between enhanced fluorescence of the amyloidogenic dye and protein concentration (Fig. 1A), while the fluorescence emission maxima remained constant at 483 nm regardless of protein concentration (Figure S2). Furthermore, after aeration atomic force microscopy (AFM) revealed the presence of rodlet-shaped assemblies on a dried down droplet of SC16 in water on highly oriented pyrolytic graphite (HOPG) (Fig. 1B). No assemblies were observed by AFM in the absence of aeration prior to drying on HOPG at the deposited concentration tested. The ~ 2 nm height of the rodlet assemblies is similar to that reported for SC3 (2.5–3 nm)^24^. The distribution of rodlet widths is comparatively narrower than the distribution of lengths and is centred at approximately 5 nm (Fig. 1C,D). The length distribution is centred at approximately 15 nm.

### SC16 adopts a unique hydrophobin structure

**Figure 2:**
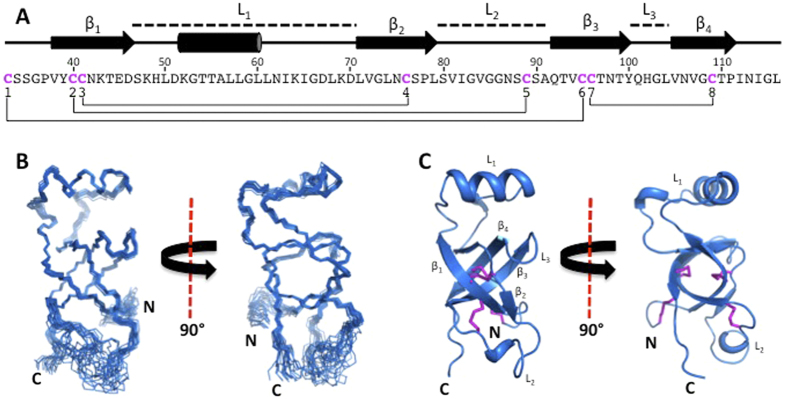
Solution structure of SC16. (**A**) The sequence of SC16 from the first cysteine residue to the C-terminus. The secondary structure is indicated for each residue and the locations of the loops, β-sheets and helix of SC16 are indicated. Cysteine residues are coloured fuchsia and disulphide linkages are indicated by a line. (**B**) Superposition of the 20 lowest-energy structures of SC16, with only the N, C^α^, and C’ atoms shown. (**C**) A ribbon diagram of the lowest-energy SC16 structure. Disulphide bonds are shown in fuchsia and the β-sheets and loops are indicated.

Detailed structural characterization of SC16 was performed by NMR spectroscopy. Multi-dimensional, heteronuclear spectra of SC16 were of high quality with well-dispersed resonances allowing for the assignment of 84%, 80% and 100% of backbone, side chain and aromatic ^1^H, ^15^N, and ^13^C resonances, respectively (Figure S3A). Due to chemical exchange and intrinsic disorder, a subset of resonances corresponding to Thr18-Asn25 were not assignable while resonances from residues Gly26-Leu116 were nearly completely assigned. An ensemble of twenty SC16 structures was determined using automated assignment with ARIA and displayed good overall structural statistics and low pairwise root mean squared deviation values (Fig. 2 and Table S1). SC16 comprised four β-strands (β_1_: Val38-Asp46 β_2_: Leu71-Leu79 β_3_: Gln92-Tyr100 β_4_: Val105-Pro111) and an α-helix (Lys52-Leu60; Fig. 2B). β_1_ and β_2_ formed a short antiparallel β-sheet linked by an ordered loop (L_1_, also referred to as the Cys3–4 loop) that contained the α-helix. β_3_ and β_4_ formed a second antiparallel β-sheet connected by a β-hairpin motif (L_3_, Cys7–8). A disordered loop (L_2_) connected β_2_ to β_3_. For our discussion of hydrophobin structure, L_1_, L_2_, and L_3_ are considered to be the intervening sequences between the four core β-strands, regardless of the specific structures they adopt. The four β-strands assembled into a β-barrel with a hydrophobic core. A secondary buried hydrophobic region in SC16 existed between the surface of the β-barrel and a hydrophobic face of the α-helix present in L_1_. SC16 adopted an ordered, compact globular structure (17 Å × 17 Å × 35 Å) with the exception of the N-terminal region comprising Lys10-Ser32, L_2_, and Ile112-Leu116 at the C-terminus, which {^1^H}-^15^N heteronuclear NOE measurements indicated to be dynamic on the ps-ns timescale (Figure S3B). The smaller dimensions of the SC16 monomer (17 Å) are similar to the AFM measured height of SC16 rodlets (~20 Å). The eight cysteine residues of SC16 formed disulphide bonds in a pattern consistent with that observed in other hydrophobins. Two disulphide bonds were located within the β-barrel of SC16 and covalently linked β_1_ to β_2_ (Cys41-Cys76) and β_3_ to β_4_ (Cys96-Cys109). The two remaining disulphide bonds connected the N-terminal tail to β_3_ (Cys33-Cys95) and β_1_ to L_2_ (Cys40-Cys89).

Using the POPS (Parameter OPtimized Surfaces) algorithm^25^ on the core region of SC16 (Cys33-Leu116), we determined that 53% of the solvent accessible surface area of SC16 was hydrophobic. Analysis of the surface charge distribution of SC16 revealed several acidic, basic, and uncharged patches (Figure S4) but the latter, in particular, was not as prominent for SC16 as for EAS, DewA and MPG1^8^,^19^,^20^. Closer inspection revealed this surface to comprise several backbone amide groups and side chain hydroxyl groups but lack hydrophobic side chain groups. This observation is unusual as other Class I hydrophobins typically display large exposed hydrophobic regions, which have been hypothesized to be responsible for mediating self-assembly at hydrophobic interfaces^8^,^19^,^20^.

**Figure 3:**
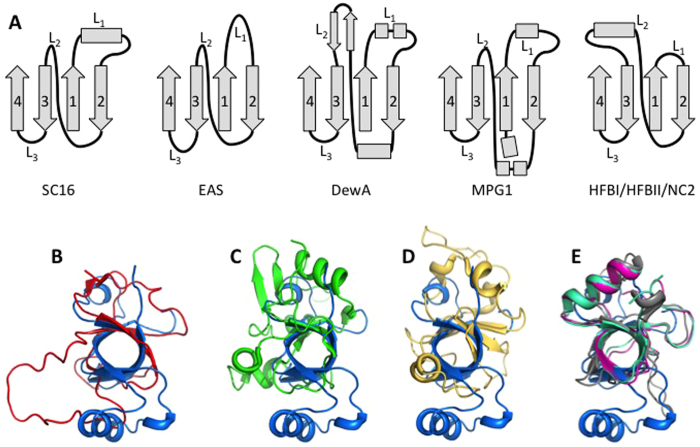
SC16 structural features are reminiscent of Class II hydrophobins. (**A**) Schematics of the secondary structure elements of SC16, the Class I hydrophobins (EAS, DewA and MPG1), and the Class II hydrophobins (HFBI, HFBII, and NC2). Both β-sheets (1-4) and intervening loops (L_1_-L_3_) are indicated. Superposition of the β-sheet core of SC16 (blue) with the Class I hydrophobins EAS (**B**; red), DewA (**C**; green), and MPG1 (**D**; yellow) and (**E**) the Class II hydrophobins HFBI, HFBII, and NC2 (purple, teal, and grey, respectively).

Of the structural features displayed by SC16, some were consistent with those previously observed in hydrophobins while others were unique. SC16 maintained the key features of the hydrophobin fold: a central four-strand β-sheet and four disulphide bonds (Fig. 2)^8^,^19^,^20^,^21^,^22^,^23^. Notably, in solution its sheet adopted a compact β-barrel structure more similar to the core structures of the Class II hydrophobins HFBI and HFBII (backbone atom root mean squared deviation of 3.0 Å and 2.2 Å, respectively; Fig. 3E) than to those of other Class I and II hydrophobins. These, in contrast to SC16, were observed as either open or irregular barrel structures (Fig. 3A–D).

Structural features shared between SC16 and the Class II hydrophobins HFBI, HFBII, and NC2, were particularly notable. Specifically, all four proteins consist of a β -barrel with an associated α-helix. However, the location of the α-helix differs in the two proteins. In SC16 it is located within L_1_ while for the Class II hydrophobins it resides in L_2_. This difference results in the α-helix being situated on opposite sides of the β-barrel when SC16 is compared to the Class II hydrophobins. Furthermore, the α-helices of the Class II hydrophobins are covalently linked to the core β-barrel through disulphide linkages, while in SC16 a hydrophobic interface is formed between the α-helix and β-barrel. This lack of a disulphide bond in SC16 may allow a greater degree of conformational flexibility to adapt to a structure amenable to rodlet formation; a posit consistent with the observation that SC16 is an active Class I hydrophobin forming rodlets under conditions of aeration.

Comparing the secondary structural elements surrounding the β-sheet core of the structurally characterized hydrophobins, it becomes apparent that the regions connecting the β-sheet strands (L_1_–L_3_) are variable in sequence, length, structure, and dynamics amongst members of the protein family (Fig. 3). In SC16, these connecting loops are most structurally similar to those of the Class II hydrophobins. As described above, in Class II, L_2_ contains an α-helix while L_1_ and L_3_ are very short. In contrast, the corresponding regions in the Class I hydrophobins (EAS, DewA, and MPG1) are much more structurally diverse and the extent of dynamics within the loop regions vary. In EAS, L_1_ and L_3_ are large and unstructured and L_2_ contains a β-strand. In MPG1 these long loops contain α-helical regions and in DewA they include both α-helices and β-strands (Fig. 3). Considering these regions within SC16, L_1_ and L_3_ are ordered while they are flexible in EAS^8^. In DewA all three loops appear well ordered^19^, while in MPG1, only L_1_ displays conformational flexibility^20^. The L_2_ region is dynamic only in SC16.

The NMR-derived secondary structure of the hydrophobin RodA^26^ has been recently reported and it has some similarities with the structure of SC16. In both RodA and SC16 a four-strand β-sheet is observed, and an α-helix is present in L_1_ while L_2_ appears to be largely disordered. There are potential structural differences between these proteins as well since L_3_ is predicted to be disordered in RodA while in SC16 it is incorporated into the β-strands. Finally, an α-helix is predicted to be present in the N-terminal region of RodA that is not found in SC16.

### Rodlet formation mechanism of SC16

Hydrophobin rodlet formation mechanisms have been refined based on the structures of EAS^8^,^27^, DewA^19^, and MPG1^20^. In recent models, the L_3_ loop is proposed to undergo a structural transition to a β-strand that oligomerizes with the L_3_ of other monomers to form amyloid rodlets^27^. While the dimensions of the SC16 rodlets measured by AFM are consistent with this model, the L_3_ of SC16 is a β-turn that connects β_3_ and β_4_, and is thus unavailable for amyloidogenesis. Only the L_1_ and L_2_ regions of SC16 are long enough to potentially undergo the conformational changes required for rodlet formation. Interestingly, the Waltz algorithm^28^ predicts that residues 40–47 of L_1_ in SC16 are amyloidogenic. Although our data indicate this loop is not dynamic and is folded into an α-helix, it may still possibly undergo structural rearrangement. Similar structural rearrangements have been observed for the closely related hydrophobin SC3, which forms a transient α-helical structure before undergoing further structural transitions to form a β-sheet rich rodlet^29^. Currently, it is not known whether a single loop region is responsible for amyloidogenesis in all known structures. Although this phenomenon has been linked to L_3_ in EAS, L_3_ is not likely to initiate such an assembly in SC16 or DewA, while in MPG1 the role of L_3_ is unclear.

In contrast to SC3^12^, SC16 only forms rodlets upon aeration. This finding is similar to MPG1, where agitation is necessary for rodlet assembly as it occurs via a surface driven mechanism. This discrepancy could simply be due to variation of protein concentrations and buffer conditions in our studies since rodlet assembly of SC3 is both concentration and buffer dependent^12^. Intriguingly, L_1_ of SC16 buries ~100 Å^2^ of hydrophobic surface area that could become accessible if L_1_ were to undergo a structural rearrangement when positioned at an interface, allowing for amyloidogenesis. Finally, glycosylation of SC3 was demonstrated to contribute to the kinetics of assembly, stability of solution monomer, and stability of films^30^. Based on primary sequence, SC16 is predicted to have fewer glycosylation sites^31^. These insights suggest that minor sequence differences or glycosylation status may be responsible for fine-tuning the functions of hydrophobins.

### SC16 is a representative member of a Basidiomycota Class I hydrophobin subdivision (Class IB)

To contextualize the structural and functional properties of SC16, we constructed a database of confirmed and predicted hydrophobin sequences from the genomes and transcriptomes of 215 unique filamentous fungi (72% Basidiomycota, 28% Ascomycota). The bias towards the Basidiomycota phylum is a reflection of the available genomic and transcriptomic data as Ascomycota species are comparatively understudied^32^. A total of 1046 canonical sequences were identified as predicted or confirmed hydrophobins. Using the PFam database of protein families^33^, 781 sequences were assigned as Class I (PFam01185), and 215 to Class II (PFam06766), while 50 were not predicted to belong to the hydrophobin family, including the well-characterized Class I EAS. PFam classifications of other previously biophysically characterized hydrophobins are consistent with their experimentally determined classes^10^.

**Figure 4:**
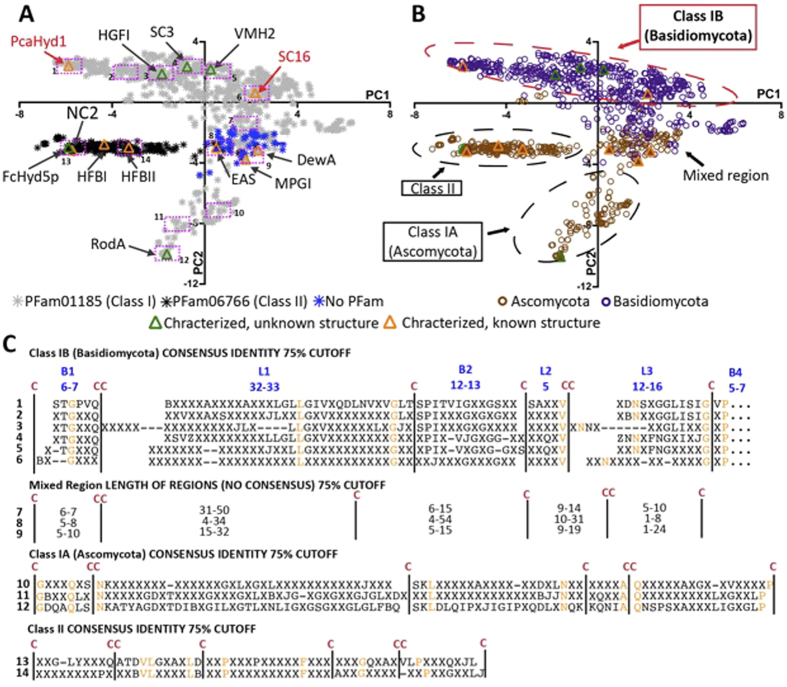
Clustering of hydrophobin sequences in the most significant components of a principal component analysis (PCA) of the sequence alignment matrix of 1052 confirmed and predicted hydrophobin sequences. PFam prediction of Classes is indicated by colour of symbol fill in (**A**) while phylum is indicated by colour of symbol fill in (**B**). Hydrophobins whose structures have been resolved are indicated in orange: EAS (*Neurospora crassa*), MPG1 (*Magnaporthe grisea),* DewA (*Aspergillus nidulans*), NC2 (*Neurospora crassa*), HFBI and HFBII (*Trichoderma reesei*). Hydrophobins whose structures remain unknown but have been characterized at various levels are indicated in green: SC3 (*Schizophyllum commune*), VMH2 (*Pleurotus ostreatus*), RodA (*Aspergillus fumigatus*) FcHyd5P (*Fusarium culmorum*). (**C**) Consensus sequences generated from the alignments of the sequences located within the 14 equal-sized pink rectangles in (*A*).

To more comprehensively assess the differences and similarities between hydrophobin sequences, we conducted a principal component analysis (PCA) of the sequence alignment matrix of the canonical set (Fig. 4A,B). Briefly, sequences that cluster together along a principal component tend to share similarities, the degree of similarity increasing with distance from the origin. The first principal component (PC1) describes the most significant differences in the data while the differences described by PC2 are less significant in the context of the total data, but could prove to be more significant for a subset of sequences.

In this analysis, both phylum and PFam prediction of hydrophobin class correlate with position in the PC2 vs. PC1 plot (Fig. 4A,B). Class II hydrophobins are readily distinguished as they form a distinct cluster, a result consistent with the established high sequence conservation of the class^17^. In contrast, class I hydrophobins reside in all four quadrants of the plot displaying, as expected, comparatively high sequence variability^7^. In PC2, however, there is a distinct separation of Ascomycota and Basidiomycota Class I sequences, with an intermediate region containing sequences originating from both phyla consistent with previous phylogenies^17^,^18^,^34^. The mixed-region of Class I contains all previously structurally characterized Class I hydrophobins and has little sequence or loop length conservation, an observation consistent with the current description of the Class I group of hydrophobins in the literature^11^.

The furthest clusters from the origin (i.e., twice as far as any other in PC2) are Class I Ascomycota (Class IA) sequences that include the *Aspergillus fumigatus* RodA. This suggests these sequences diverge from those of other hydrophobins in high identity clusters, a result consistent with previous phylogenies^18^. However, what is particularly unique about this analysis is the emergence of a highly conserved Class I subdivision consisting of Basidiomycota sequences (Class IB). Furthermore, this subdivision includes the experimentally confirmed Class I sequences HGFI (*Grifola frondosa*), VMH2 (*Pleurotus ostreatus)*, along with the *S. commune* SC3 and SC16.

To further appreciate the sequence-based clustering of Class I hydrophobins, we generated alignments of the sequences located within 14 equal-sized regions across PC2 vs. PC1 (Fig. 4C; full alignments in Figure S6A–AB, tables of identities in Table S2A-L). Consistent with the clustering of Class IB in the PCA, the 6 consensus sequences from Class IB hydrophobins contain similar loop lengths and a number of conserved residues in addition to the cysteine pattern throughout the hydrophobin sequence. The Class IA and Class II hydrophobins were also observed to have similar loop lengths and additional conserved residues within their groups. In contrast, very little sequence conservation is observed for the mixed region. This suggests that hydrophobins within the Class IA and IB regions may have similar structural and functional properties to other members of their respective groups.

To validate this hypothesis, we collected structural data on another predicted Class IB hydrophobin PcaHyd1 (*Phanerochaete carnosa)*, which is located at the opposite side of the Class IB sequence cluster from SC16. Using NMR spectroscopy, we completed the backbone ^1^H, ^13^C, and ^15^N chemical shift assignments for PcaHyd1 and calculated the chemical shift index for each residue of the protein^35^. The predicted secondary structure elements of PcaHyd1 aligned with the structured regions of SC16 (Figure S5, which is consistent with the high sequence conservation within the core regions of SC16 and PcaHyd1 (42% and 59% sequence identity and similarity, respectively). Overall, it is likely that SC16 and PcaHyd1 share the same three-dimensional fold, which would be common to Class I Basidiomycota hydrophobins given the high sequence similarity observed for this Class.

All previously determined Class I hydrophobin structures (i.e., EAS^8^, DewA^19^, and MPG1^20^) display distinct structural features from each other and cluster in the mixed region of the PCA analysis, which contains large sequence variations. This suggests that these structures are not broadly applicable to Class I sequences. In contrast, SC16 is representative of the Class IB hydrophobins, given the high sequence identity of the subdivision and similar structures of SC16 and PcaHyd1. Such an integrated structure-bioinformatic analysis will lead the way to characterizing the sequence determinants of hydrophobin function and improve our understanding of functional amyloidosis.

## Materials and Methods

### Bioinformatic analysis of hydrophobin sequences

The UniProt, JGI, and NCBI databases were mined for the canonical eight cysteine pattern residue: > = Cys to the left of the first CC, > = Cys between the two Cys, and > = Cys to the right of the second CC (see SM for more details). The resulting FASTA sequences were submitted to signal IP using default parameters to determine the predicted cleavage site of the secretion signal and to PFam^33^ for domain assignation. Sequence alignment of the resulting 1046 mature predicted and confirmed hydrophobins was constructed using MAFFT (multiple alignment using fast fourier transform)^36^. Using this sequence alignment, Jalview (vs 2.8.2)^37^ was used to produce a principal component analysis of the sequence alignment matrix using default parameters.

### Plasmid construction, protein expression and purification

DNA coding for residues Thr18-Leu116 of the *Hyd1* (SC16) gene of *Schizophyllum commune* or residues Thr21-Leu138 of the *Hyd1* gene of *Phanerochaete carnosa* were cloned into a pET32 vector downstream of DNA coding for His_6_-tagged thioredoxin (Trx) and an enterokinase cleavage site. The N-terminal signal peptide was not cloned and after cleavage the amino acids KAMADIGS remain at the N-terminus of this SC16 construct. Cultures of Origami B (DE3) *E. coli* were grown in LB media supplemented with 15 μg/mL kanamycin, 12.5 μg/mL tetracycline, and 100 μg/mL carbenicillin at 37 °C until the culture reached an O.D. 600 of 0.6–0.8. Protein expression was induced with 300 μM isopropyl β-D-thiogalactopyranoside and occurred for 20 hours at 23 °C.

After expression, cell pellets containing Trx-SC16 were lysed in lysis buffer (20 mM Tris-HCl pH 8, 250 mM NaCl), clarified by centrifugation and the Trx-SC16 protein was isolated via Ni^2+^ affinity. His_6_-tagged Enterokinase was added in a ratio of 1 unit per 500 μg of protein to remove the Trx tag, and the sample was dialyzed against 20 mM Tris-HCl pH 8, 150 mM NaCl, and 2 mM CaCl_2_. After protease cleavage, the Trx and enterokinase were separated from SC16 by a second round of Ni^2+^ affinity chromatography and then concentrated using ultrafiltration. Purified protein was either used immediately for experiments or lyophilized in 20 mM ammonium acetate at pH 4.5 and stored in a desiccator for long-term storage. The observed molecular weight of purified SC16 corresponded to its calculated molecular weight of 10.8 kDa. The PcaHyd1 protein was prepared using the same procedure as SC16. For NMR sample preparation the *E. coli* culture was grown in M9 minimal medium supplemented with 1 g/L ^15^NH_4_Cl, 2 g/L ^13^C-glucose, and 10 mL/L of ^13^C/^15^N-BioExpress-1000 medium (Cambridge Isotope Laboratories).

### Thioflavin T binding assay

SC16 was resuspended in 20 mM phosphate buffer (pH 7.5) to a concentration of 66 μg/ml. The solution was left to incubate at ambient temperature for 12 hours (referred to as “overnight shaking”) either with or without gentle mixing head over tail shaking on a rotary shaker at approximately 40 rotations per minute. After incubation, Thioflavin T was added to each solution to a concentration of 4 μM. The protein samples were then diluted as specified and emission spectra were collected (scanning between 450 and 600 nm, with 5 nm slit widths) using an excitation wavelength of 442 nm with 2 nm slit widths (Cary Eclipse Fluorescence Spectrophotometer, Varian).

### AFM studies

Freeze-dried SC16 was resuspended in milliQ water (pH4.6) at a concentration of 210 μg/ml. This solution was used to prepare a 1 ml solution at 2 μg/ml in a microfuge tube with 1 ml empty headspace. This solution was agitated using vortexer set on high for 30 minutes. A 2 μl sample from the core of the solution was then placed directly on freshly cleaved HOPG and left on the bench to dry for 30 minutes. Samples were placed in a desiccator overnight before imaging.

All atomic force microscopy (AFM) images were acquired using a Nanoscope IIIA Multimode scanning probe microscope (Digital Instruments, Santa Barbara, CA). The AFM images were collected using Nanoscope version 5.12r3 software and a J scanner that has a maximal lateral scanning area of 125 μm × 125 μm. Silicon probes TESP-V2 (Bruker AFM Probes, Camarillo, CA) were irradiated under UV light for 30 min to remove possible organic contaminants. All images were captured as 512 × 512 pixel data sets at a tip scan rate between 0.9 and 1.1 Hz with a cantilever drive frequency of between 280 and 320 kHz.

The length and width values of SC16 rodlets were determined in ImageJ (version 1.46r)^38^,^39^ using the following sequence of image processing steps: (1) unsharp filter with radius of 2 pixel and mask weight of 0.6; (2) despeckle; (3) binary threshold; (4) particle analysis using fit ellipse measurement with size from 0 to infinity and circularity from 0–0.7. The height values were determined by analyzing cross sections of the rodlets using NanoScope Analysis software version 1.50.

### NMR spectroscopy and structure calculations

NMR spectra were recorded on a Varian 600 MHz INOVA spectrometer equipped with a triple-resonance probe at 30 °C. A single protein sample containing 1 mM ^13^C/^15^N-labeled SC16 in 25 mM Tris-HCl pH 8.0 and 150 mM NaCl was used for data collection. Standard triple resonance experiments were used to assign the backbone and side-chain resonances of SC16. Distance restraints were derived from ^15^N edited NOESY-HSQC and both aliphatic and aromatic ^13^C edited NOESY-HSQC spectra collected with a 100 ms mixing time. In order to identify residues involved in hydrogen bonds, a ^1^H-^15^N HSQC spectrum was collected on a D_2_O exchanged sample of SC16. Spectra were processed using NMRPipe^40^ and analyzed using CcpNmr Analysis version 2.4^40^. Data was collected for PcaHyd1 under the same sample conditions as SC16, except that data collection occurred at 25 °C.

Backbone and side-chain chemical shift values, partially assigned NOESY peak lists and dihederal angle restraints generated by DANGLE^42^ were used as inputs to ARIA version 2.3^43^ for automated NOE assignment and structure calculation of SC16. From the initial structural folds, it was clear that SC16 contains the conserved disulphide bonding pattern of hydrophobins and these four restraints were added to the structure calculation. Hydrogen bond restraints were added for residues that have strong signal in a ^1^H-^15^N HSQC spectrum for a D_2_O exchanged sample as well as for the helical regions of the initial ensembles. The final structural ensemble of SC16 consists of the 20 lowest energy structures out of 200. These structures were refined in water by CNS 1.21^44^,^45^. The quality of the final ensemble of structures was assessed through RPF analysis^46^, PROCHECK^47^ and the PSVS suite^48^. The PyMOL Molecular Graphics System (Version 1.7.1 Schrödinger, LLC) was used for visualization, distance measurements, and figure generation. Chemical shifts and the final structural ensemble of SC16 have been deposited into the Protein Data Bank and the BioMagResBank (Accession numbers 2NBH and 25976, respectively). The chemical shift assignments of PcaHyd1 were deposited into the BioMagResBank (Accession #: 26907).

## Additional Information

**How to cite this article:** Gandier, J.-A. *et al*. Characterization of a Basidiomycota hydrophobin reveals the structural basis for a high-similarity Class I subdivision. *Sci. Rep.* **7**, 45863; doi: 10.1038/srep45863 (2017).

**Publisher's note:** Springer Nature remains neutral with regard to jurisdictional claims in published maps and institutional affiliations.

## Supplementary Material

Supplementary Information

Supplementary Dataset
