# Supplementary material for: Characterization of a Basidiomycota hydrophobin reveals the structural basis for a high-similarity Class I subdivision
Source: Sci Rep. 2017 Apr 10;7:45863. doi: 10.1038/srep45863 (PMC5385502; doi:10.1038/srep45863)
Supplement: Supplementary Information [file srep45863-s1.pdf]

Characterization of a Basidiomycota hydrophobin reveals the structural basis for a high-similarity Class I subdivision

Julie-Anne Gandier[1,*], David N. Langelaan[2,*], Amy Won[3], Kylie O'Donnell[1], Julie L. Grondin[4], Holly L. Spencer[4], Philip Wong[5], Elisabeth Tillier[5], Christopher Yip[3], Steven P. Smith[4], Emma R. Master[1,*]

[1]Department of Chemical Engineering and Applied Chemistry, University of Toronto, Toronto, Ontario, Canada
[2]Department of Biochemistry & Molecular Biology, Dalhousie University, Halifax, Nova Scotia, Canada
[3]Department of Medical Biophysics, University of Toronto, Toronto, Ontario, Canada
[4]Department of Biomedical and Molecular Sciences, Queen's University, Kingston, Ontario, Canada
[5]e-Institute of Biomaterials and Biomedical Engineering, University of Toronto, Toronto, Ontario, Canada

[*]These authors contributed equally to the work.

Correspondence and request for materials should be addressed to E.R.M. (email: emma.master@utoronto.ca)

Supplementary Materials

Supplementary Table 1: Structural statistics of the SC16 structural ensemble[a]

**Completeness of Resonance Assignments (%)[b]**	
Backbone	84.1
Side Chain	79.9
Aromatic	100
**Number of conformational restraints**	
Total NOE restraints	1495
Intraresidue (i=j)	647
Sequential (|i-j| = 1)	336
Medium range (|i-j| < 5)	167
Long range (|i-j| ≥ 5)	345
Ambiguous	51
Dihedral angle restraints	132
Hydrogen bond restraints	50
NOE Restraints per residue	14.0
Long range restraints per residue	3.2
**Residual restraint violations[c]**	
RMSD NOE restraints (Å)	0.03 ± 0.001
RMSD dihedral angle restraints (˚)	0.34 ± 0.052
Average no. of distance violations per structure	0
>0.5 Å	0
>0.3 Å	0.1
Average no. of dihedral angle violations per structure > 5˚	0.34 ± 0.05
**Model quality[d]**	
RMSD backbone atoms (Å)[e]	0.5
RMSD heavy atoms (Å)[e]	0.8
RMSD bond lengths to ideal geometry (Å)	0.005
RMSD bond angles to ideal geometry (˚)	0.8
**MolProbity Ramachandran statistics[c,e]**	
Most favoured regions (%)	92.2
Allowed regions (%)	7.4
Disallowed regions (%)	0.4
**Global quality scores (raw/Z score)[c]**	
Verify3D	0.38/-1.28
ProsaII	0.60/-0.21
PROCHECK (Φ-Ψ)[d]	-0.48/-1.57
PROCHECK (all)[d]	-0.63/-3.73
MolProbity clash score	37.12/-4.84
**RPF analysis[f]**	
Recall	0.981
Precision	0.729
F-measure	0.836
DP score	0.886
**Model contents**	
Ordered residue ranges[e]	10-116, 33-35, 37-81,91-115
Total no. of residues	107
BMRB accession number	25976
PDB ID code	2NBH

[a]Structural statistics computed for the ensemble of 20 deposited structures as recommended by the wwPDB task force.

[b]Calculating using the AVS software.

[c] Calculated using CNS version 1.21.

[d] Calculated using the PSVS version 1.5.

[e]Based on ordered residue ranges (S(f) +S(y) > 1.8).

[f]Calculated using CcpNmr Analysis version 2.3.

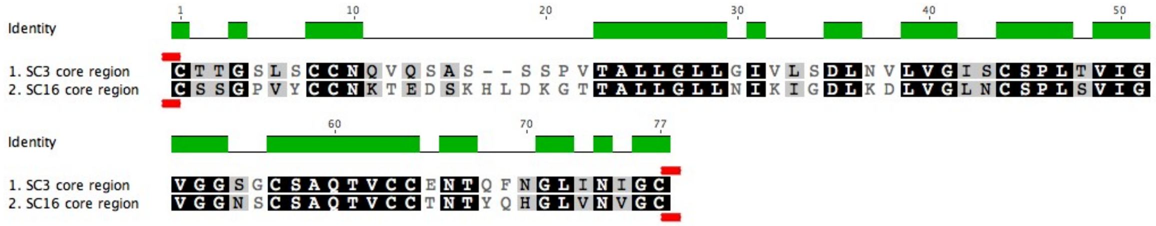

Supplementary Figure 1. Sequence alignment of the core regions of SC3 and SC16. The regions spanning from the first to last cysteines of SC3 and SC16 were aligned revealing 56% identity (ClustalW algorithm for pair-wise alignment with the Blosum cost matrix and free end gaps)

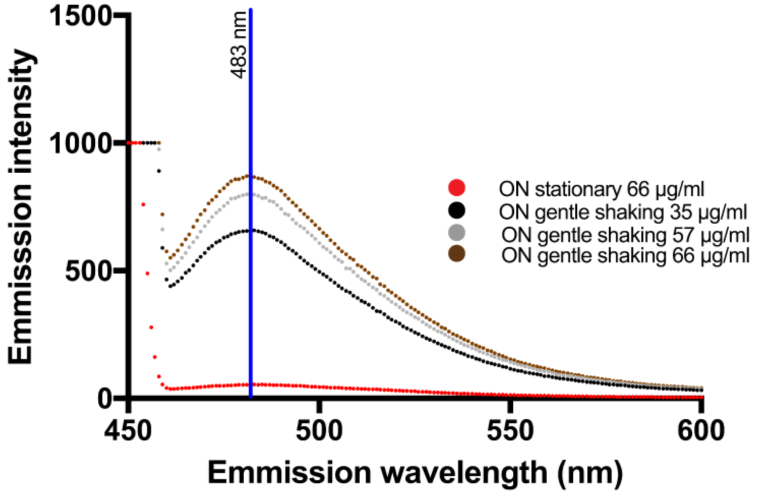

Supplementary Figure 2. Emission intensity vs. emission wavelength of Thioflavin T (ThT) excited at 442 nm . The maximum of the emission wavelength remained constant for solutions containing 35-66 µg/ml aerated overnight by gentle tail over head shaking on a rotary shaker. N=1 for each curve.

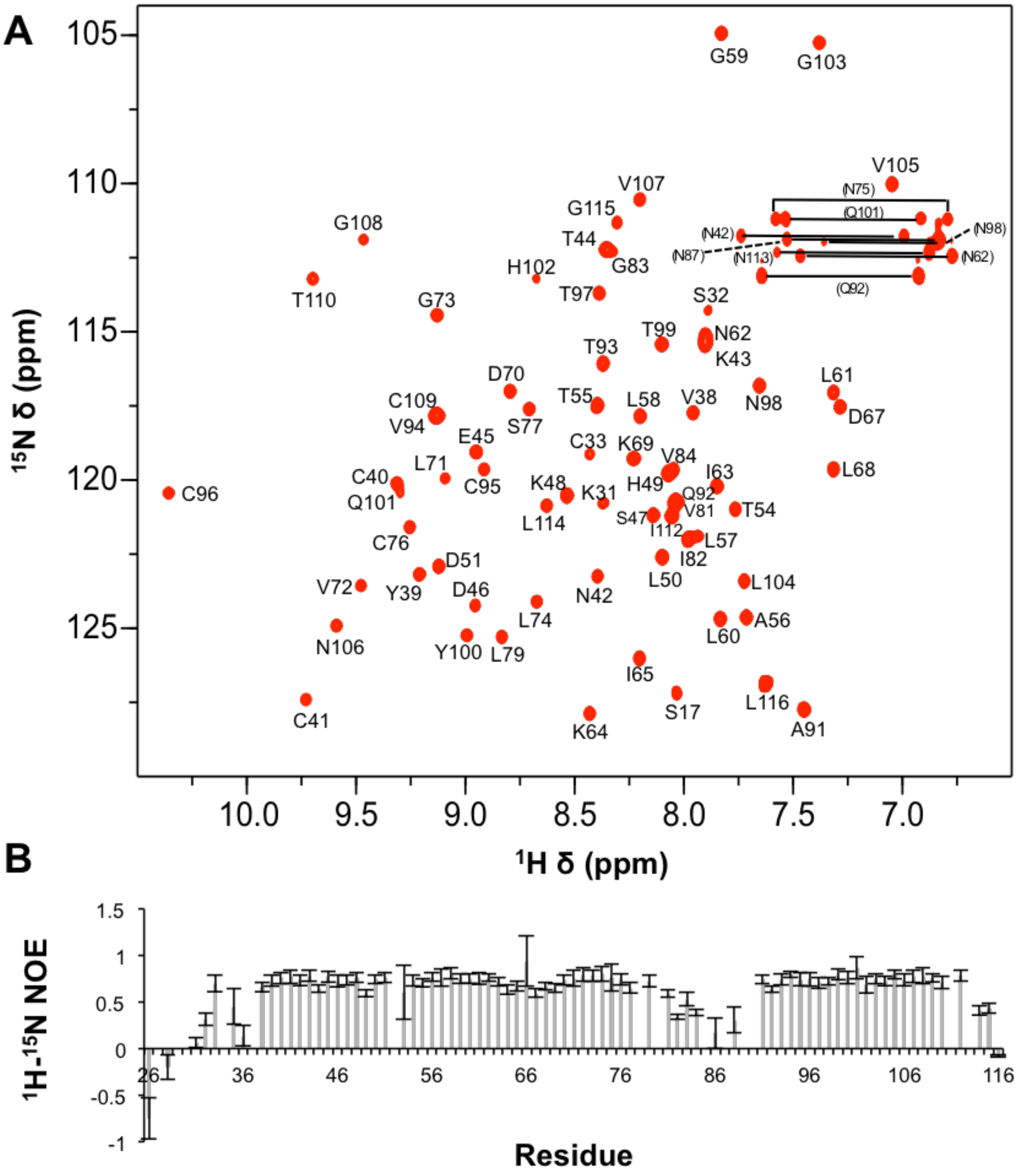

Supplementary Figure 3. SC16 adopts a well-ordered structure. **A)** A ^{1}H-^{15}N HSQC spectrum of SC16 with the assignments for backbone and side-chain resonances indicated. **B)** {^{1}H}-^{15}N NOE analysis of SC16 indicates that most of the protein is not dynamic on the ps-ns timescale.

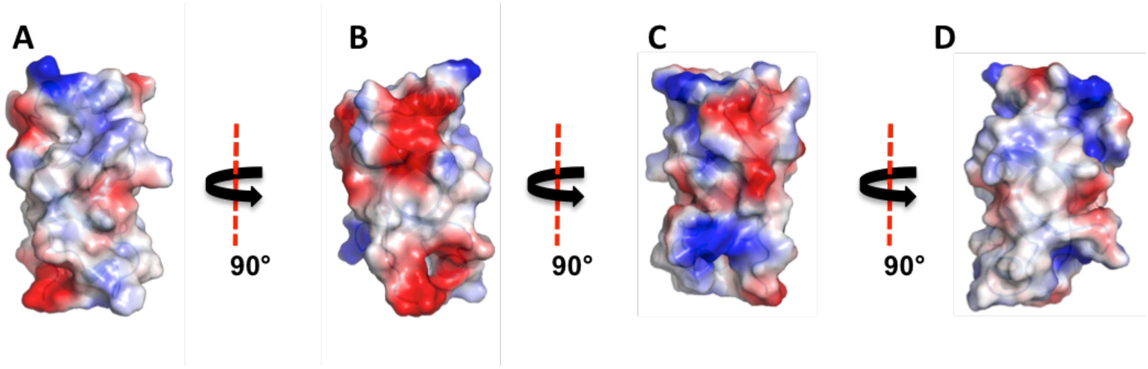

Supplementary Figure 4. The electrostatic potential surface of SC16 has several charged patches. Negative and positive electrostatic surface potentials are coloured red and blue, respectively. Although there is a large white non-charged area **(D)**, these residues are not hydrophobic in nature.

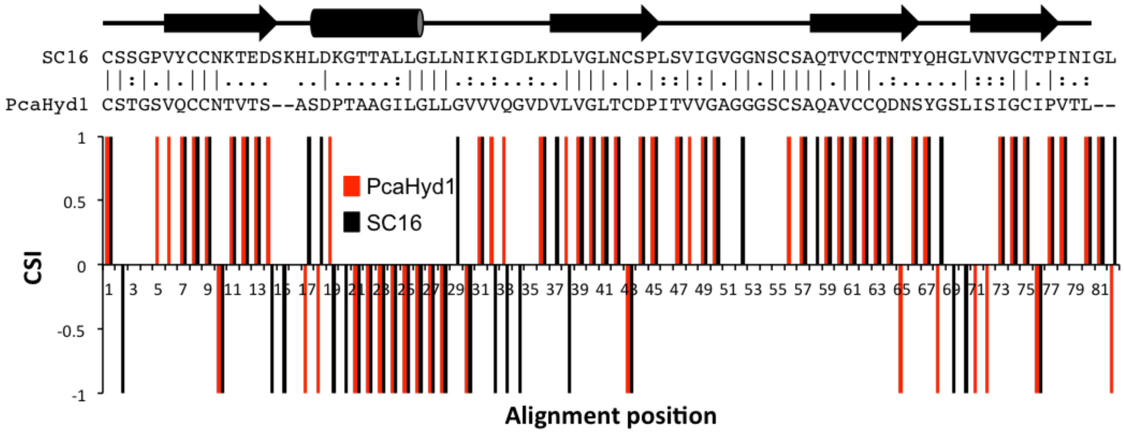

Supplementary Figure 5. SC16 and PcaHyd1 share similar secondary structures. The secondary structural elements of SC16 are indicated above the sequence alignment of SC16 and PcaHyd1. The chemical shift index based secondary structure prediction for SC16 (black) and PcaHyd1 (red) are aligned below the sequence alignment. The chemical shift index based prediction of secondary structure for SC16 and PcaHyd1 agree over well conserved regions of SC16 that are structured.

Please note in the following stacks of Supplementary Figures 6A-AB all secretion signals were removed using SignalIP as describes in the methods of the main body of the paper. Tables of identities follow the alignments.

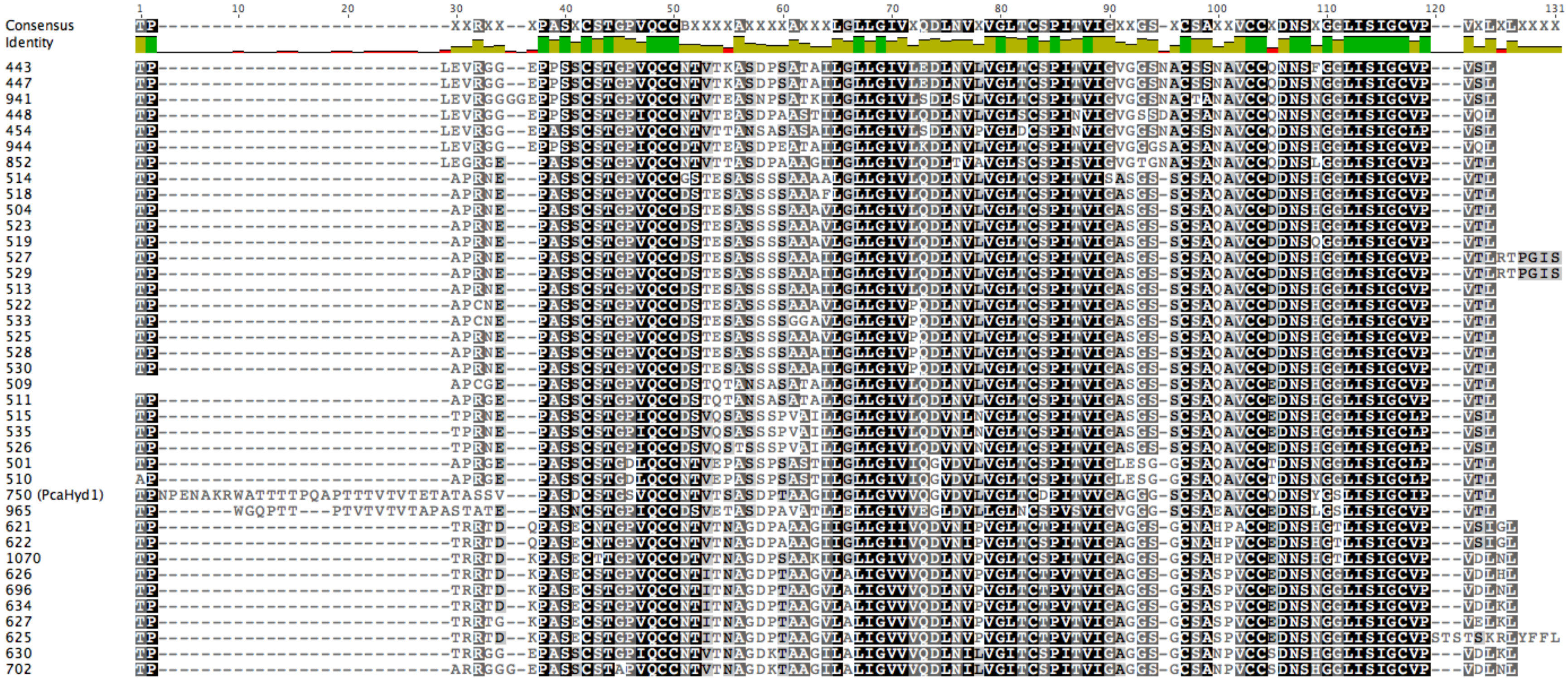

Supplementary Figure 6A. Sequence alignment and corresponding consensus sequence of proteins contained in **Region 1** (full sequence)

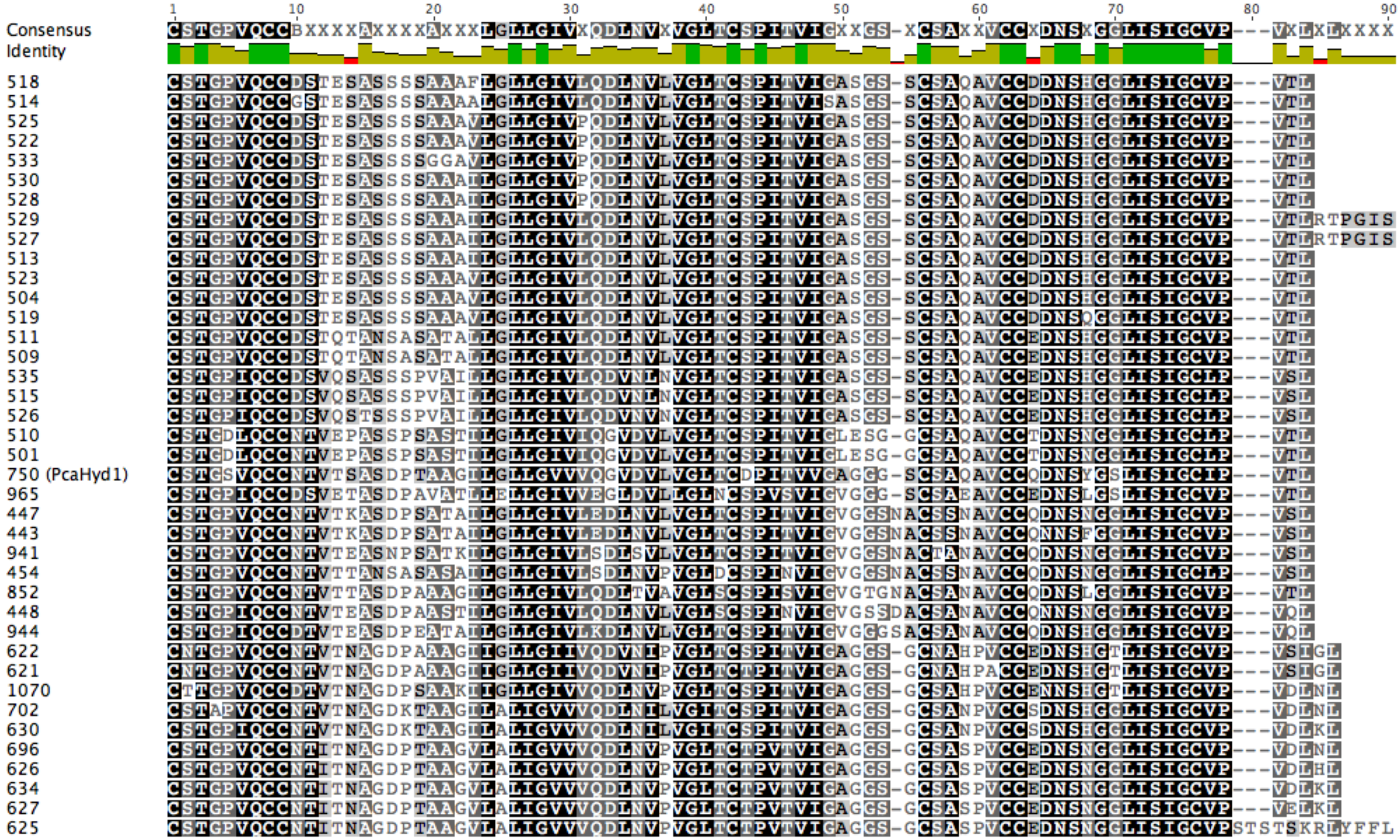

Supplementary Figure 6B. Sequence alignment and corresponding consensus sequence of proteins contained in **Region 1** (without N terminus region preceding first cysteine of the eight-cysteine pattern)

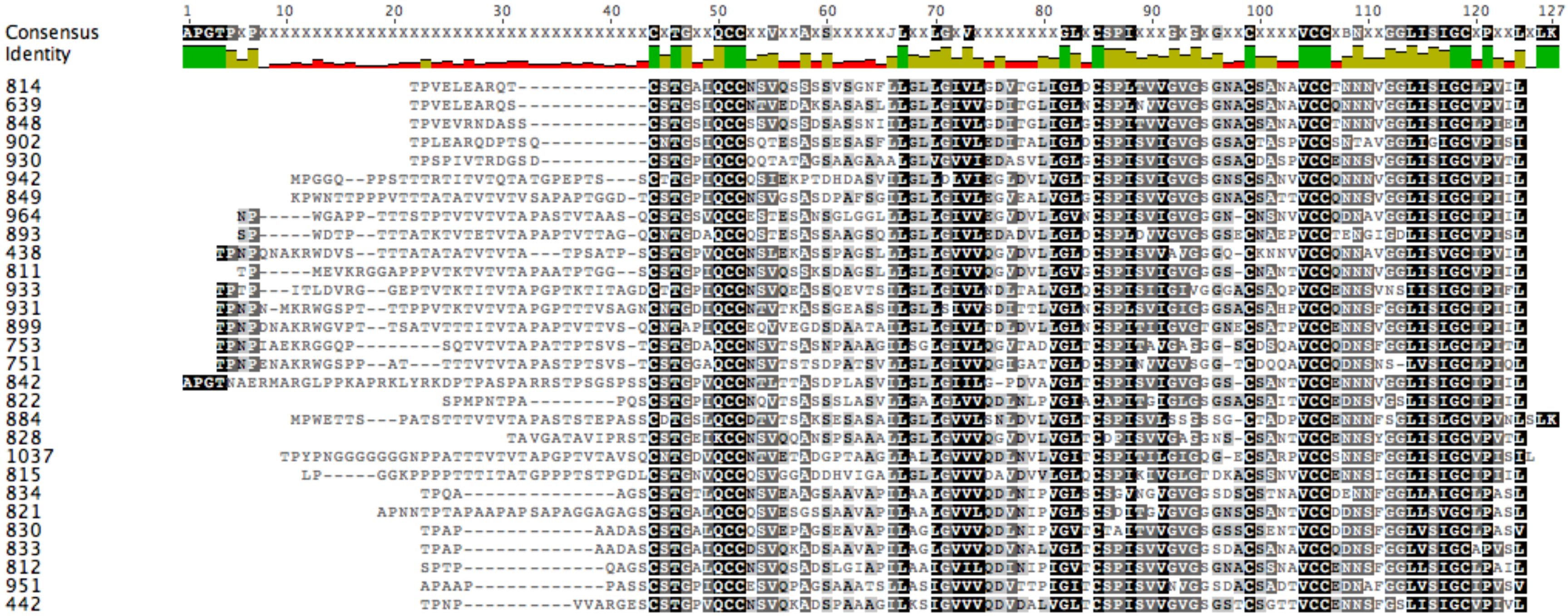

Supplementary Figure 6C. Sequence alignment and corresponding consensus sequence of proteins contained in **Region 2** (full sequence)

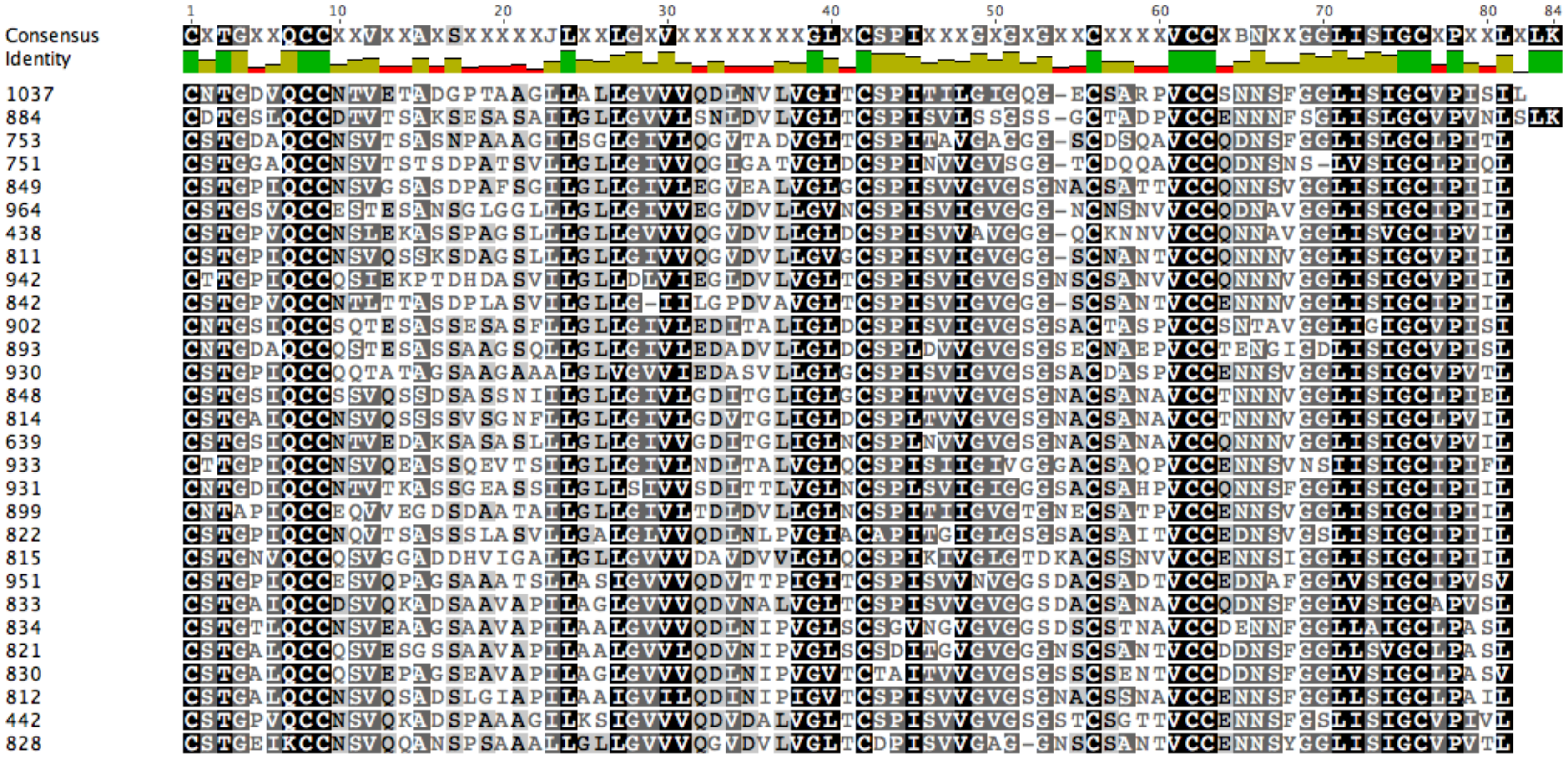

Supplementary Figure 6D. Sequence alignment and corresponding consensus sequence of proteins contained in **Region 2** (without N terminus region preceding first cysteine of the eight-cysteine pattern)

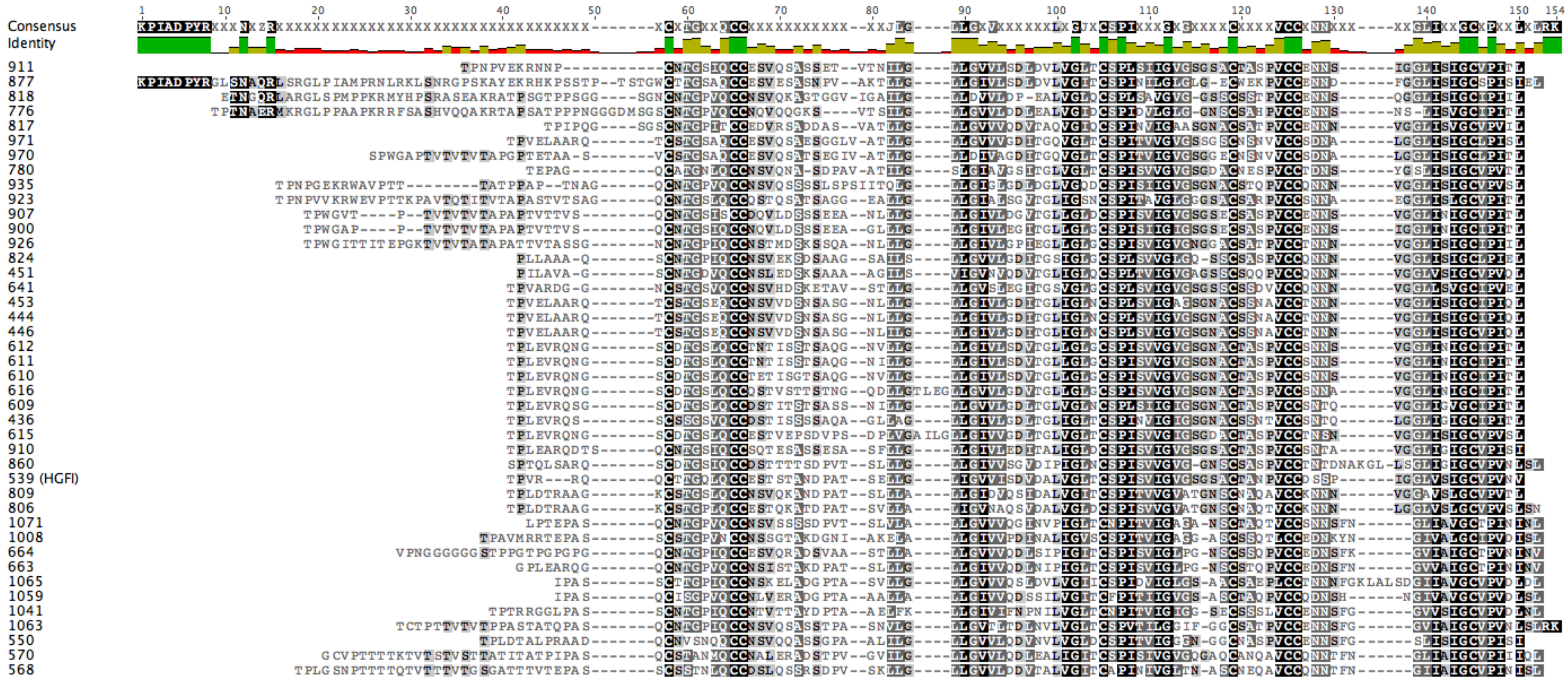

Supplementary Figure 6E. Sequence alignment and corresponding consensus sequence of proteins contained in **Region 3** (full sequence)

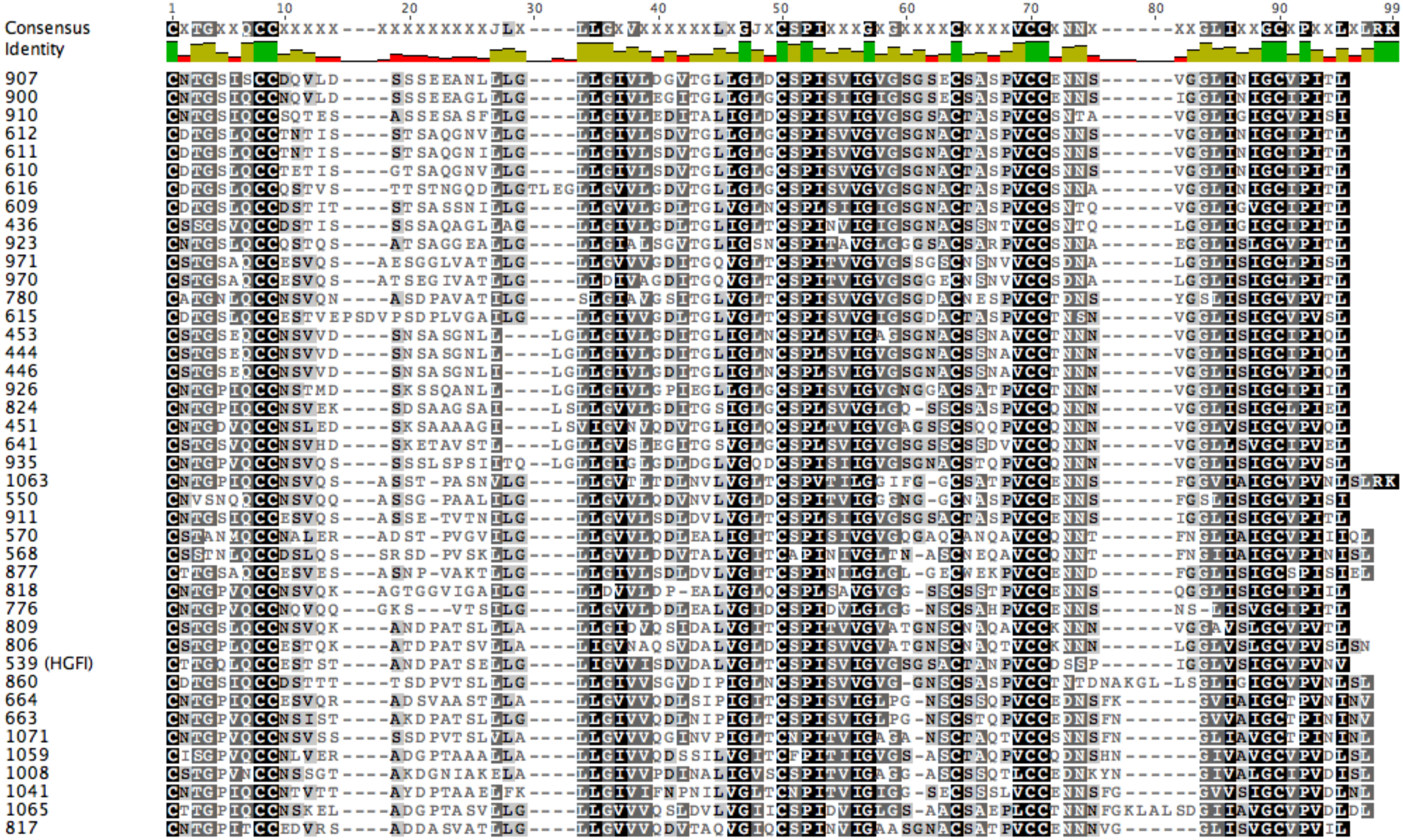

Supplementary Figure 6F. Sequence alignment and corresponding consensus sequence of proteins contained in **Region 3** (without N terminus region preceding first cysteine of the eight-cysteine pattern)

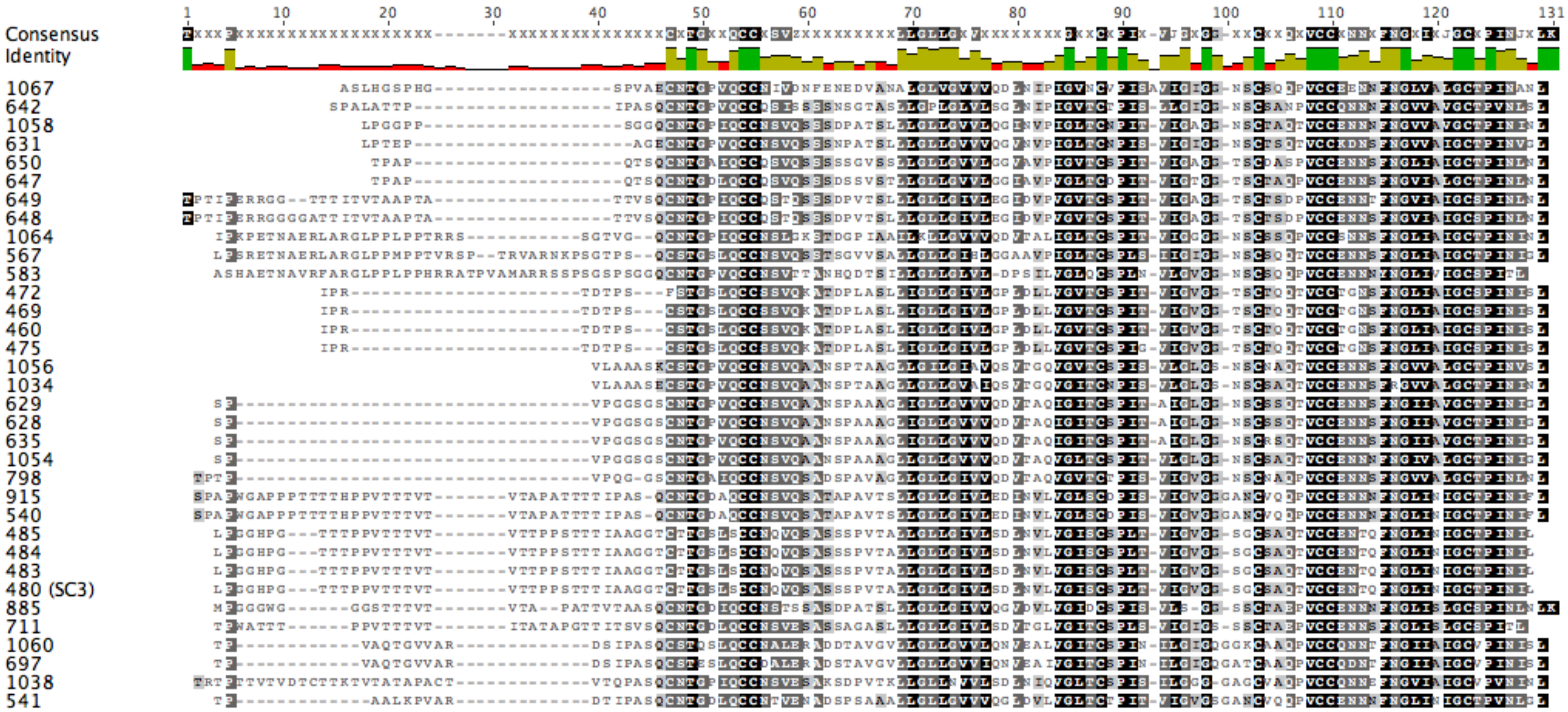

Supplementary Figure 6G. Sequence alignment and corresponding consensus sequence of proteins contained in **Region 4** (full sequence)

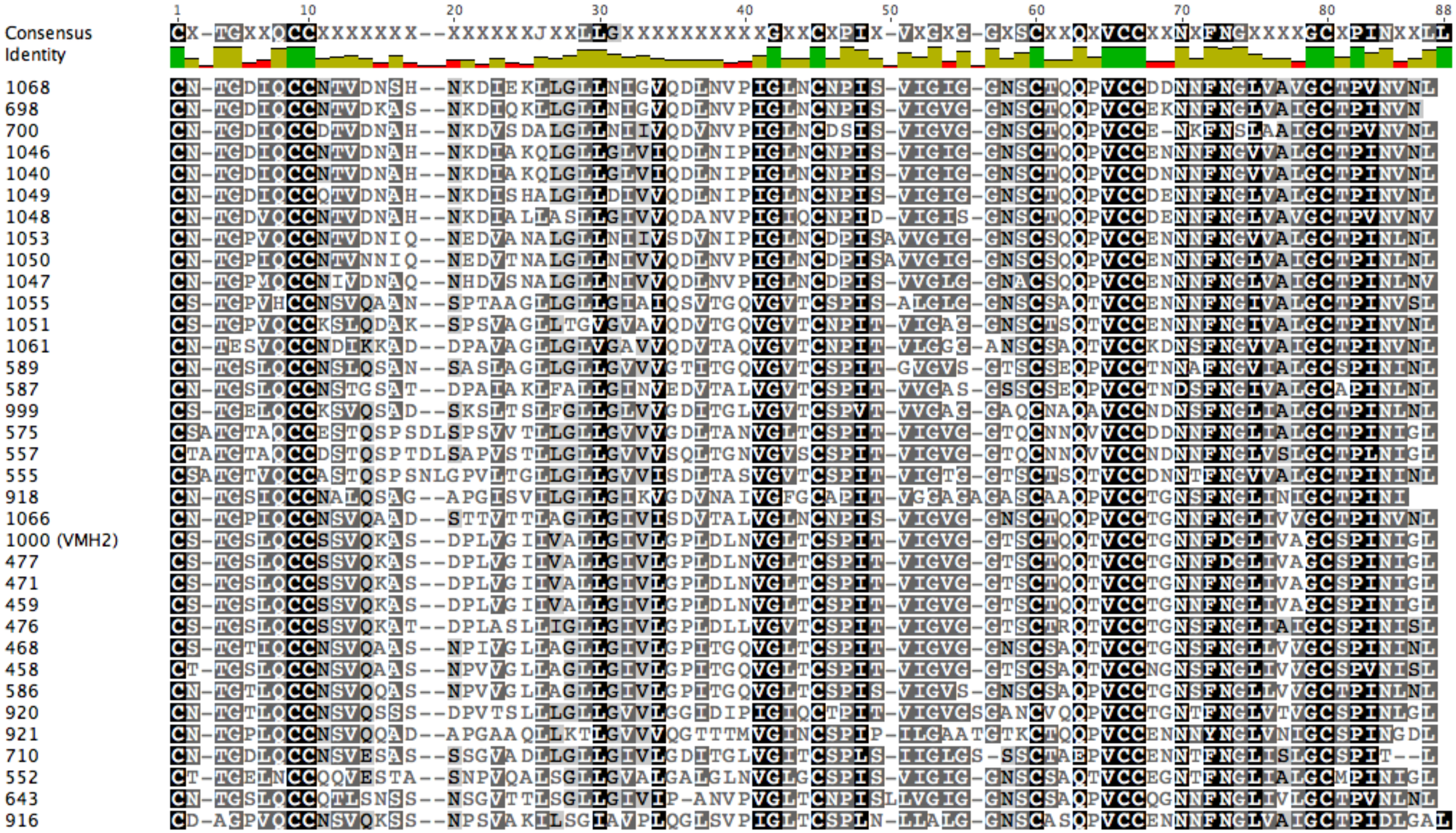

Supplementary Figure 6H. Sequence alignment and corresponding consensus sequence of proteins contained in **Region 4** (without N terminus region preceding first cysteine of the eight-cysteine pattern)

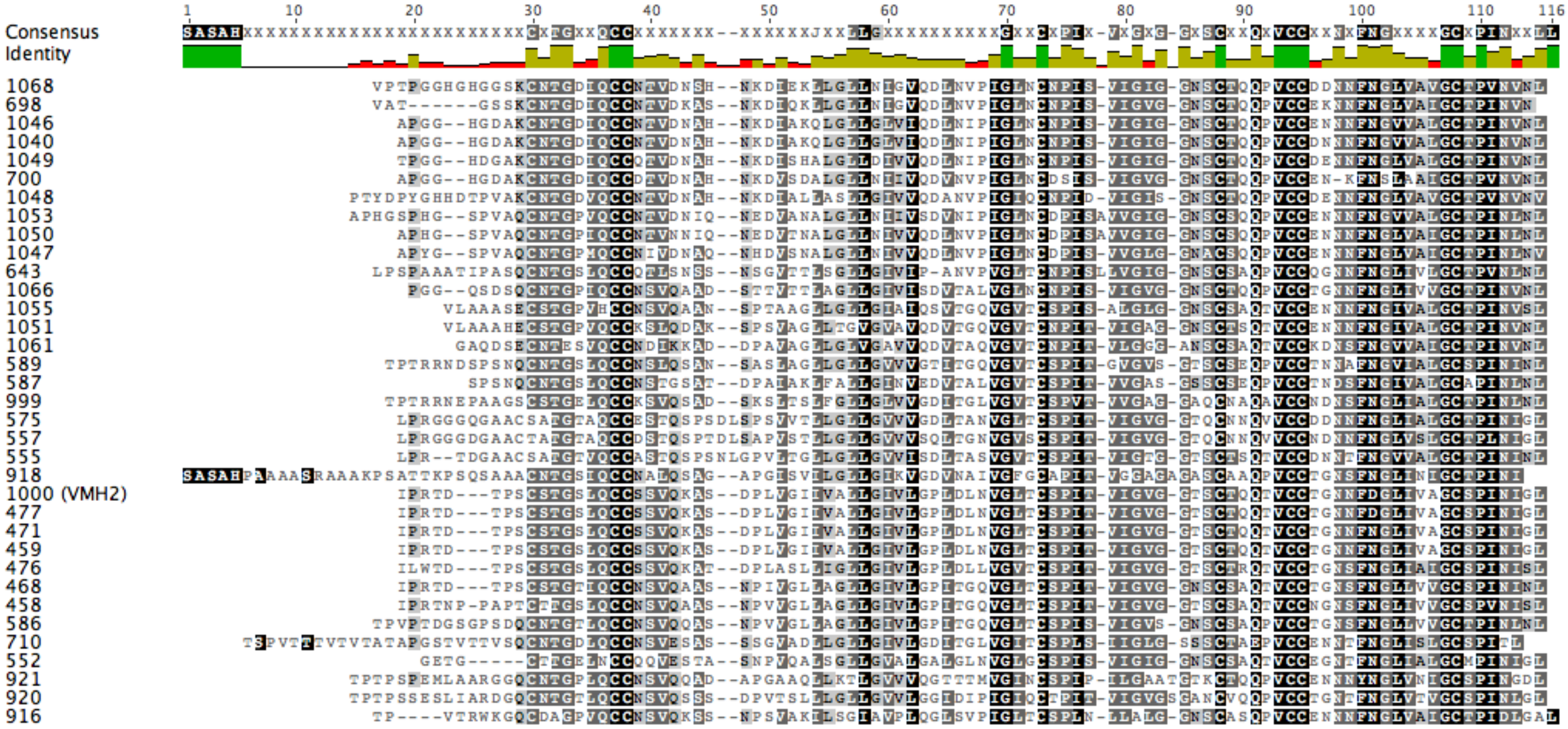

Supplementary Figure 6I. Sequence alignment and corresponding consensus sequence of proteins contained in **Region 5** (full sequence)

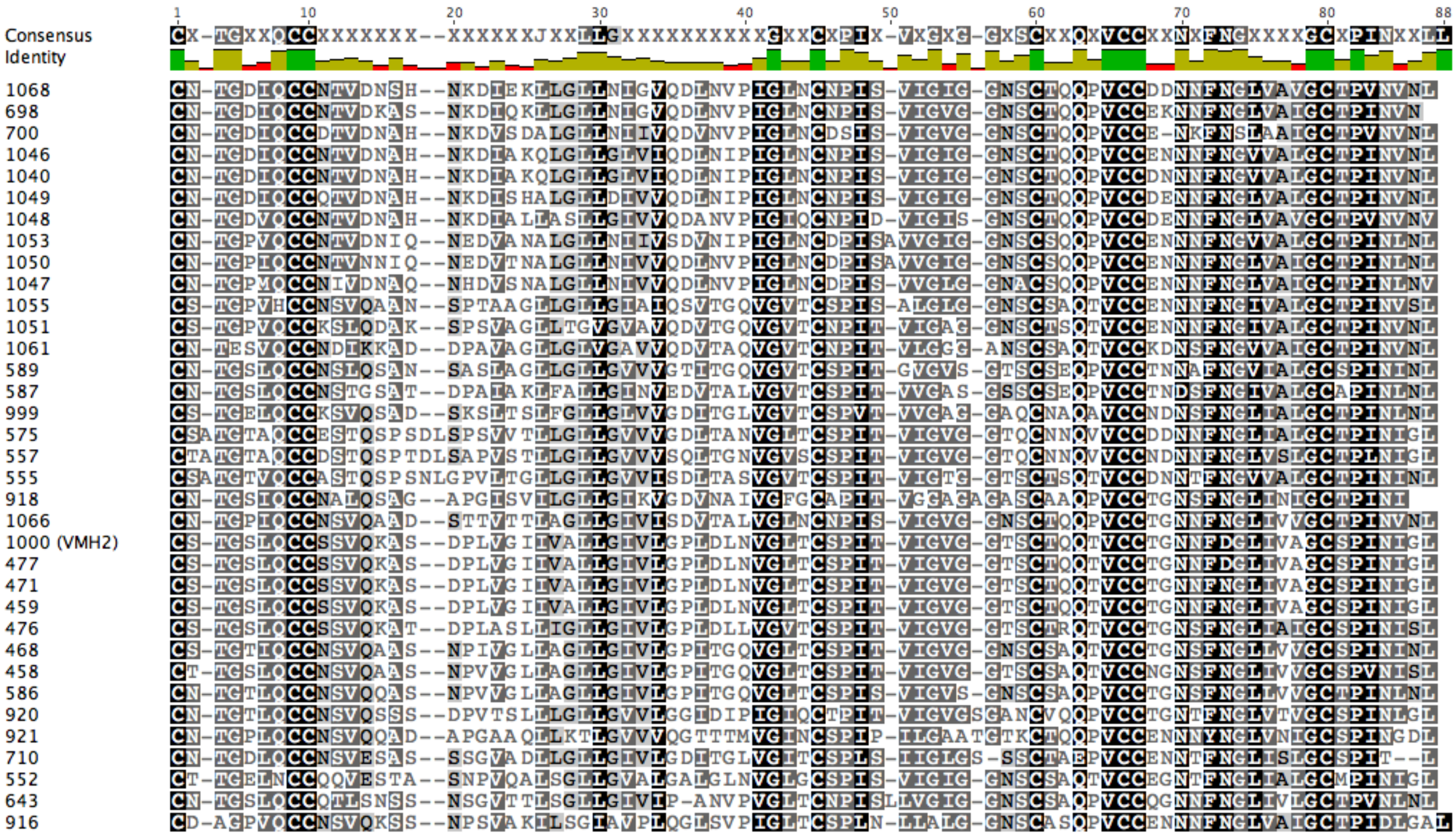

Supplementary Figure 6J Sequence alignment and corresponding consensus sequence of proteins contained in **Region 5** (without N terminus region preceding first cysteine of the eight-cysteine pattern)

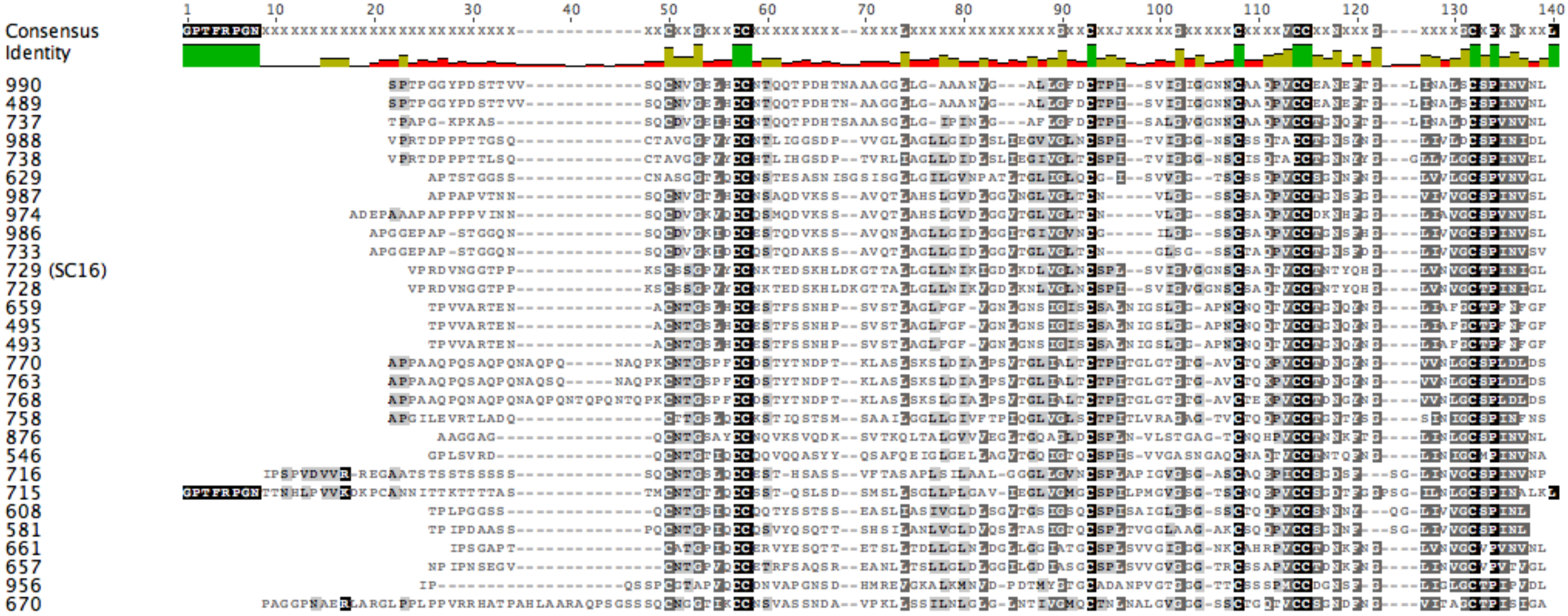

Supplementary Figure 6K. Sequence alignment and corresponding consensus sequence of proteins contained in **Region 6** (full sequence)

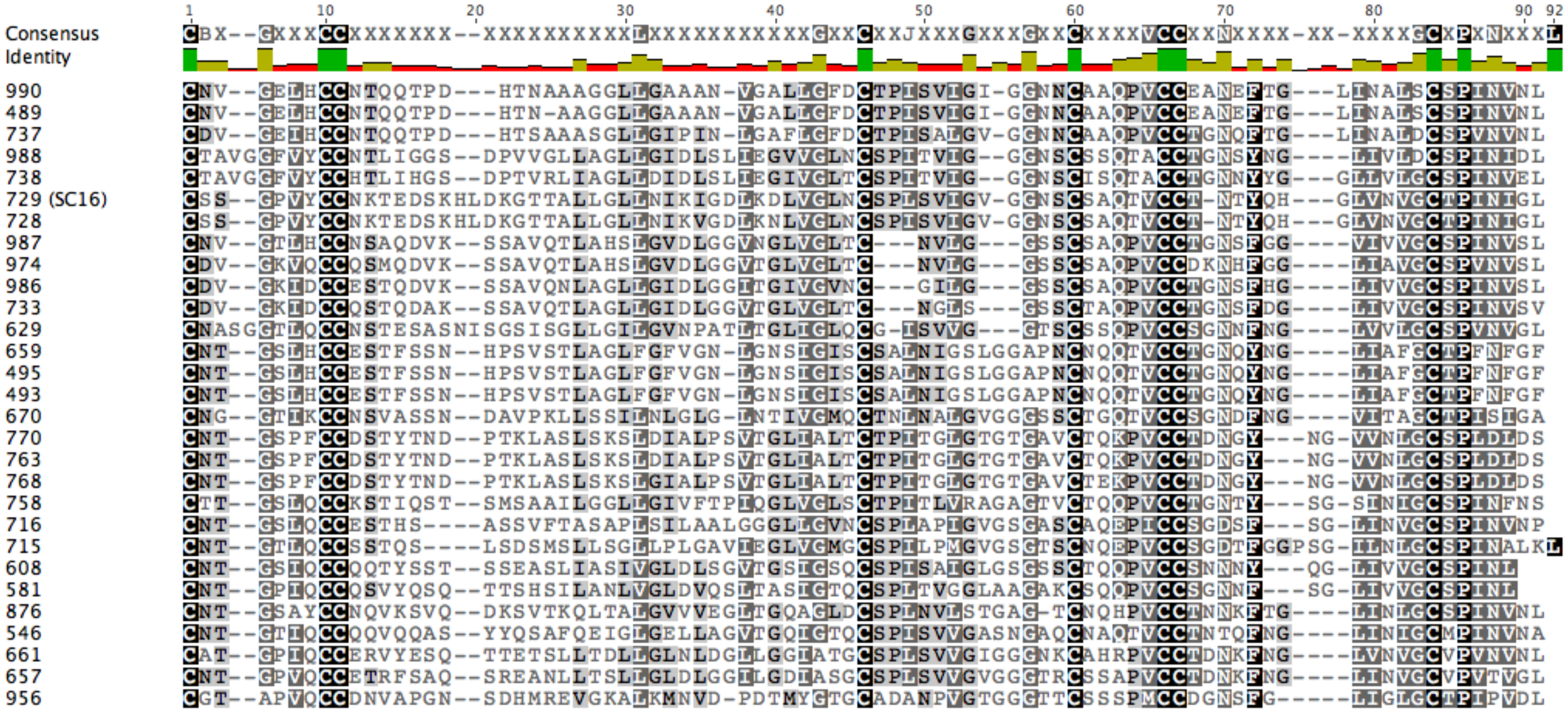

Supplementary Figure 6L. Sequence alignment and corresponding consensus sequence of proteins contained in **Region 6** (without N terminus region preceding first cysteine of the eight-cysteine pattern)

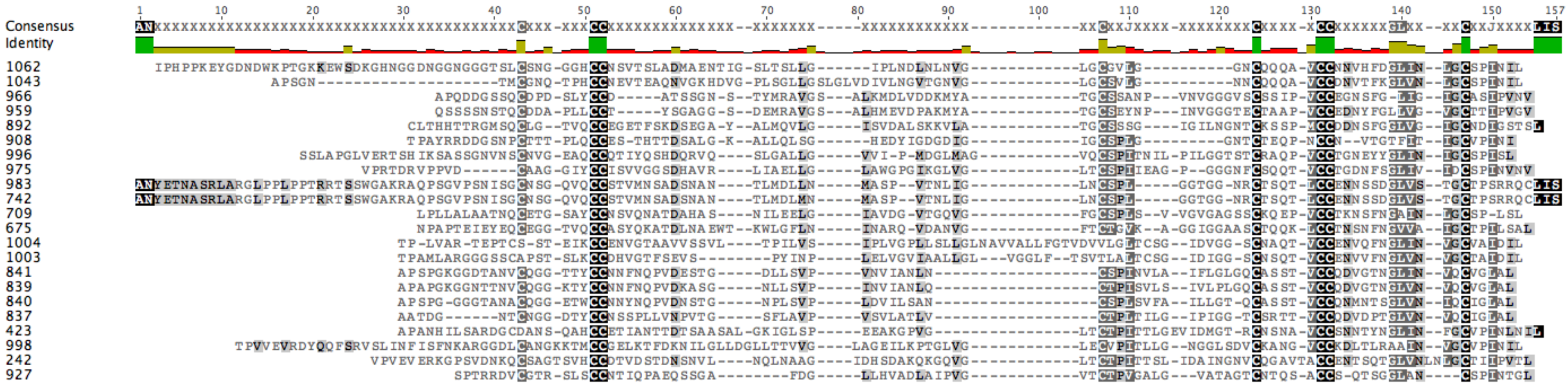

Supplementary Figure 6M. Sequence alignment and corresponding consensus sequence of proteins contained in **Region 7** (full sequence)

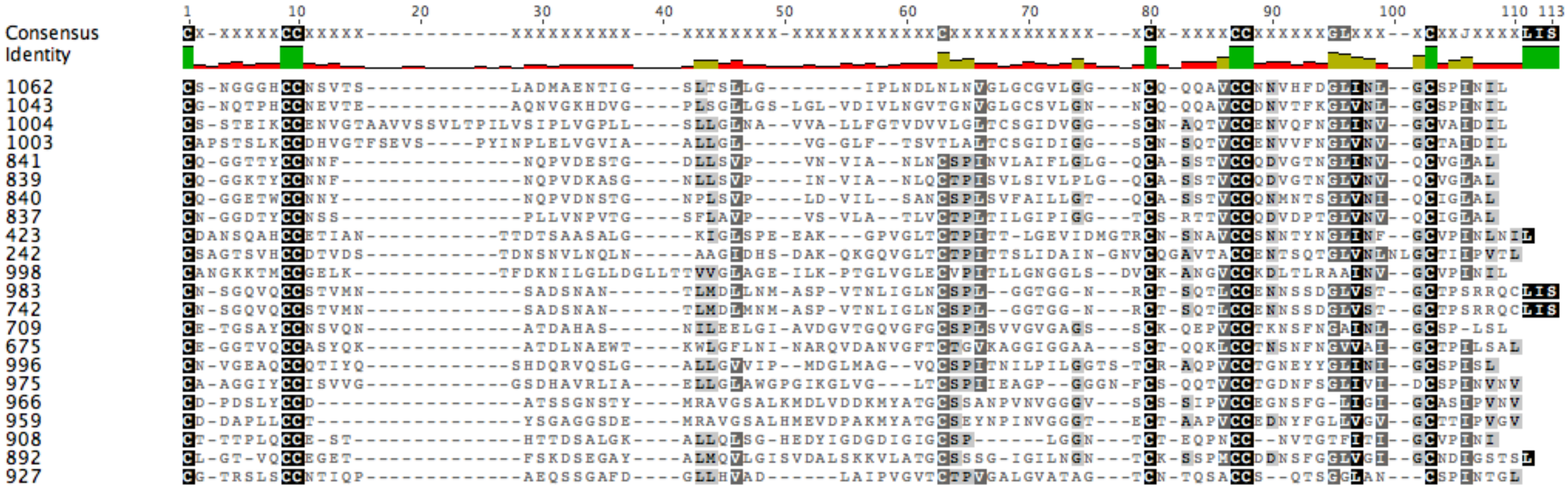

Supplementary Figure 6N. Sequence alignment and corresponding consensus sequence of proteins contained in **Region 7** (without N terminus region preceding first cysteine of the eight-cysteine pattern)

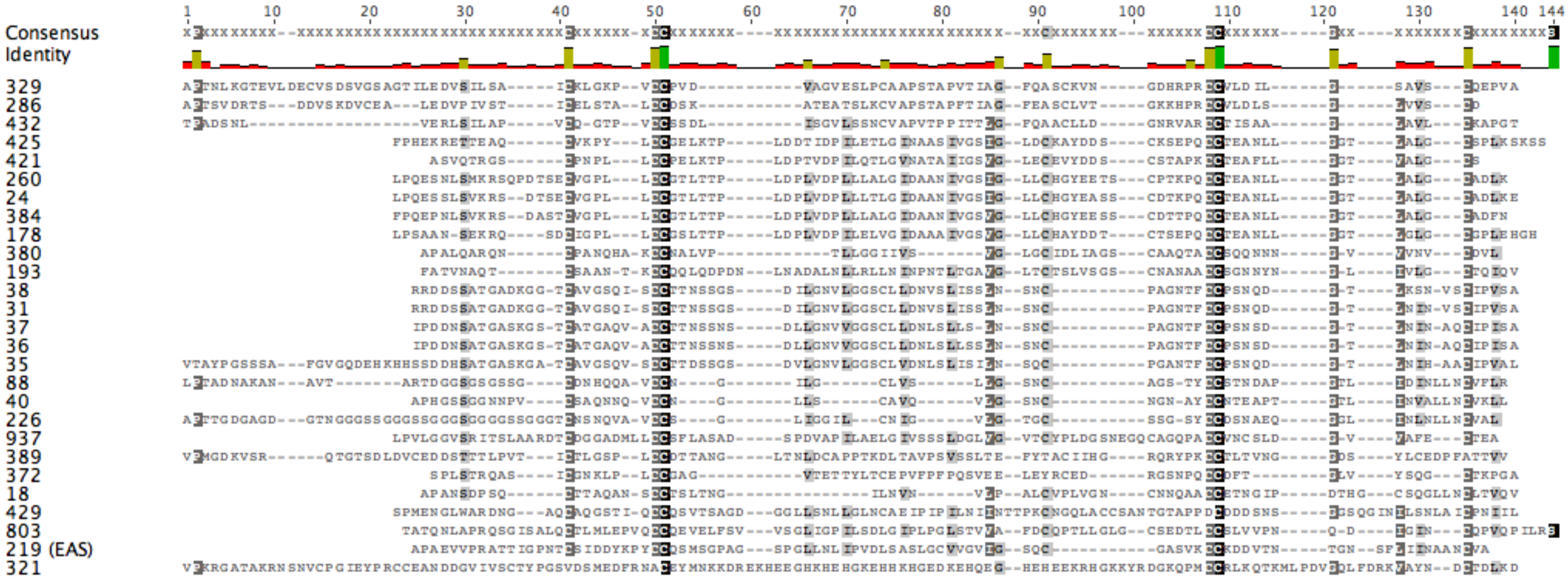

Supplementary Figure 6O. Sequence alignment and corresponding consensus sequence of proteins contained in **Region 8** (full sequence)

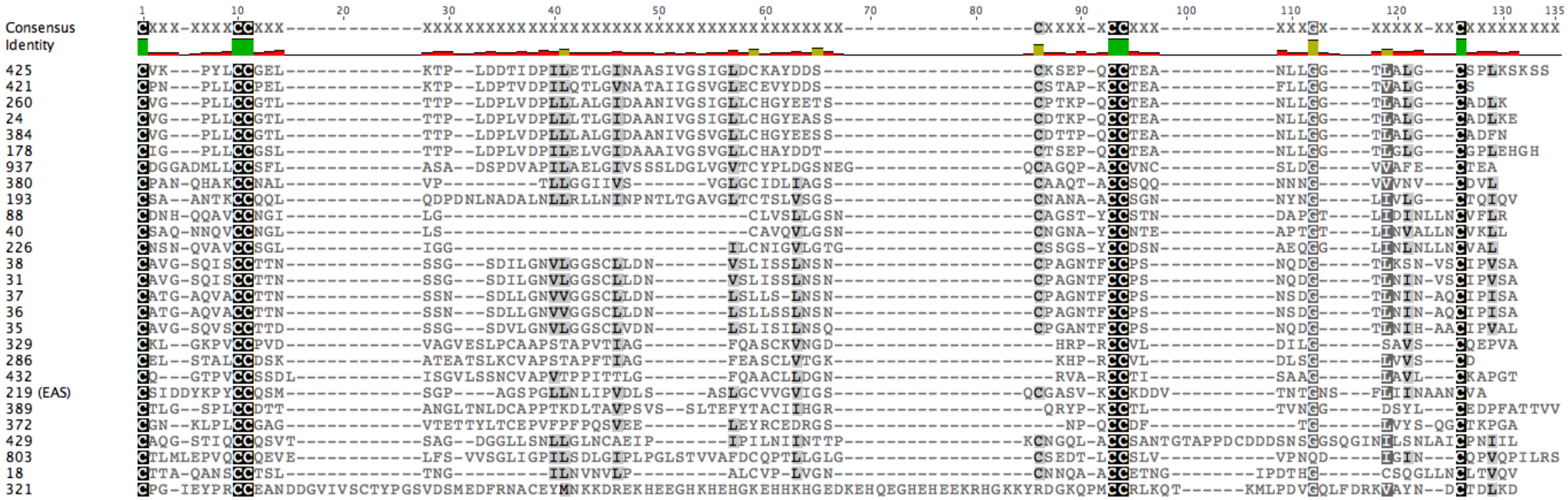

Supplementary Figure 6P. Sequence alignment and corresponding consensus sequence of proteins contained in **Region 8** (without N terminus region preceding first cysteine of the eight-cysteine pattern)

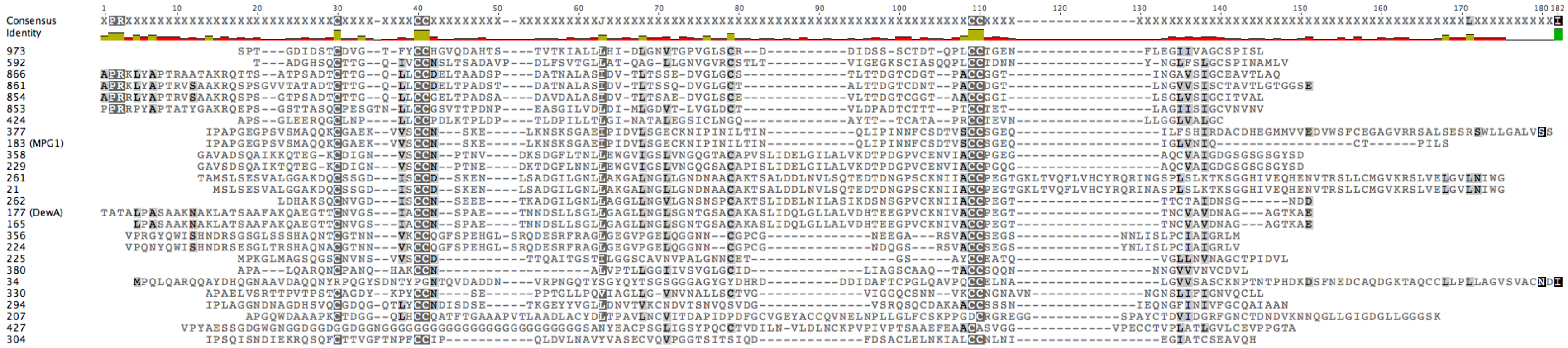

Supplementary Figure 6Q. Sequence alignment and corresponding consensus sequence of proteins contained in **Region 9** (full sequence)

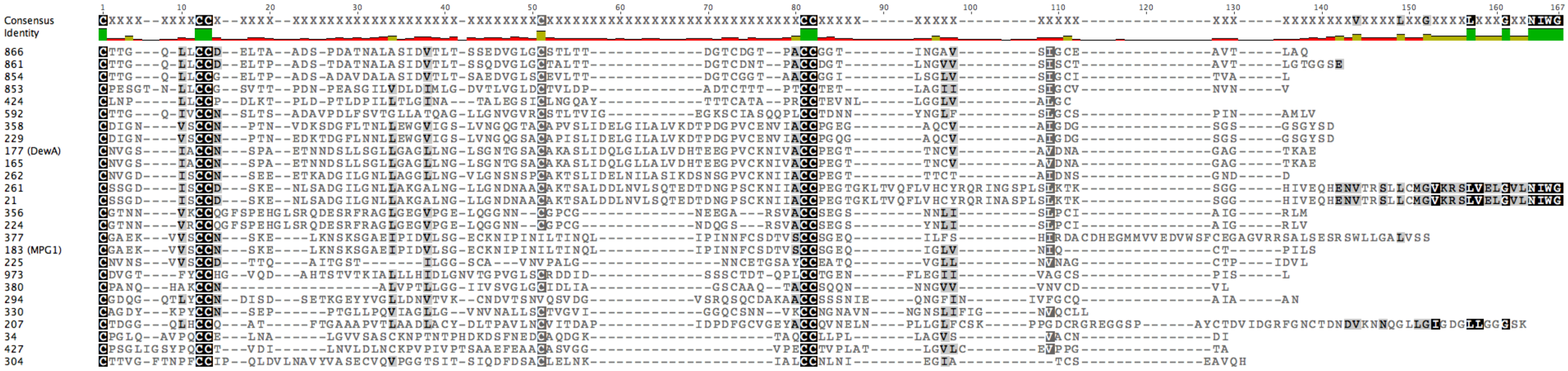

Supplementary Figure 6R. Sequence alignment and corresponding consensus sequence of proteins contained in **Region 9** (without N terminus region preceding first cysteine of the eight-cysteine pattern)

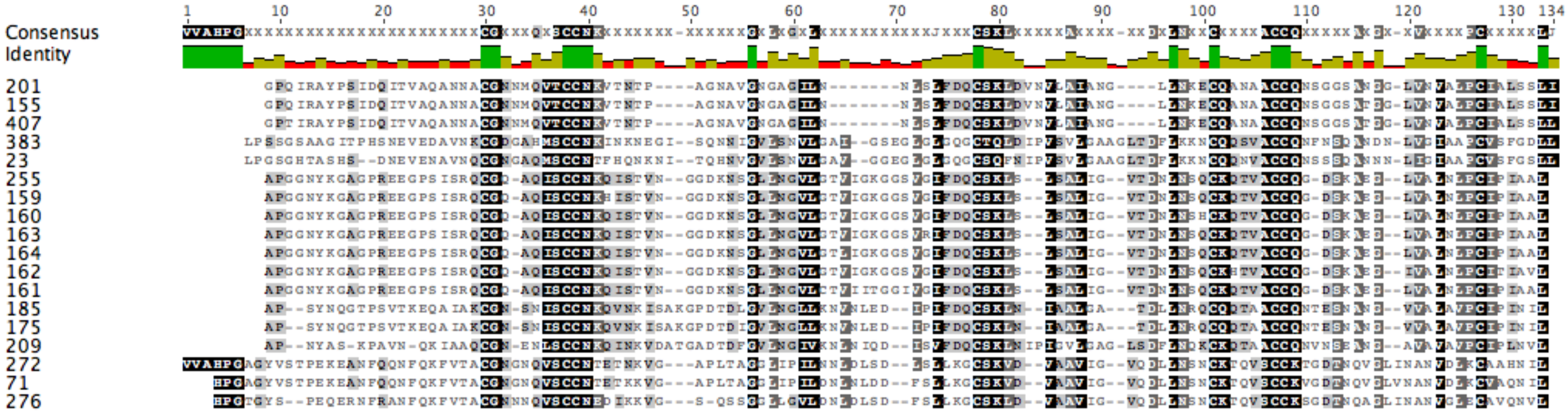

Supplementary Figure 6S. Sequence alignment and corresponding consensus sequence of proteins contained in **Region 10** (full sequence)

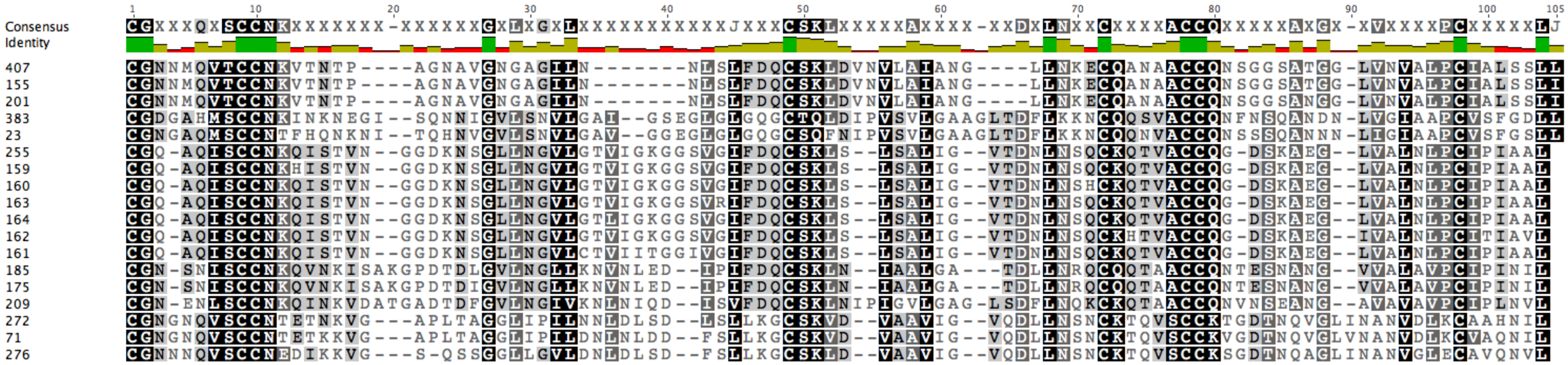

Supplementary Figure 6T. Sequence alignment and corresponding consensus sequence of proteins contained in **Region 10** (without N terminus region preceding first cysteine of the eight-cysteine pattern)

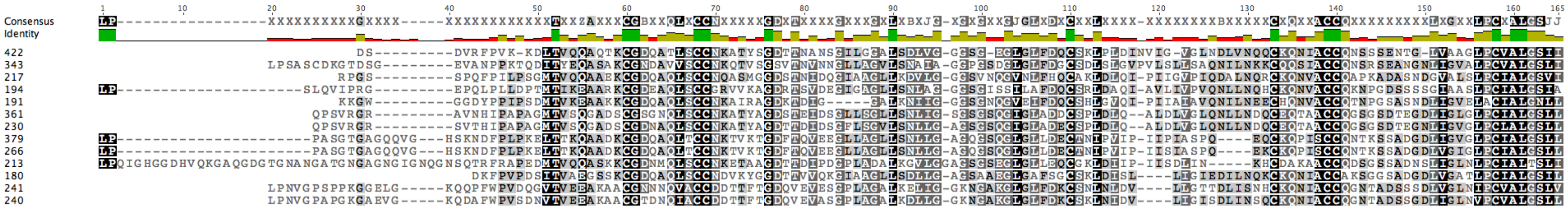

Supplementary Figure 6U. Sequence alignment and corresponding consensus sequence of proteins contained in **Region 11** (full sequence)

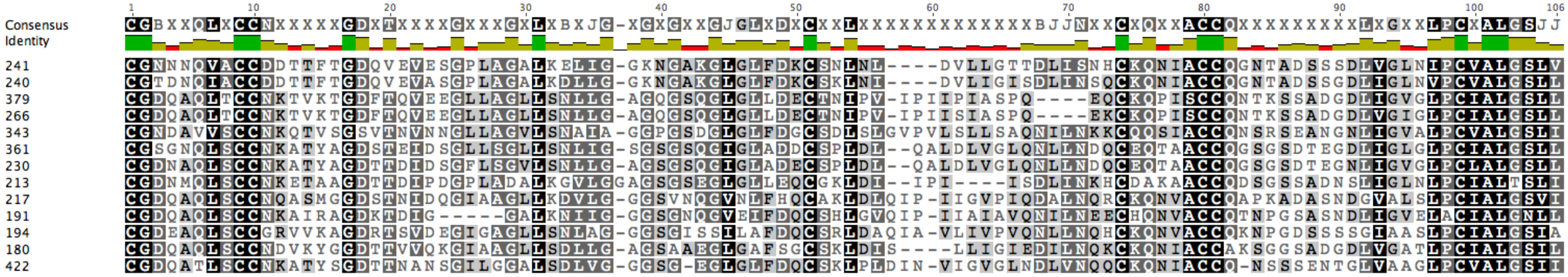

Supplementary Figure 6V. Sequence alignment and corresponding consensus sequence of proteins contained in **Region 11** (without N terminus region preceding first cysteine of the eight-cysteine pattern)

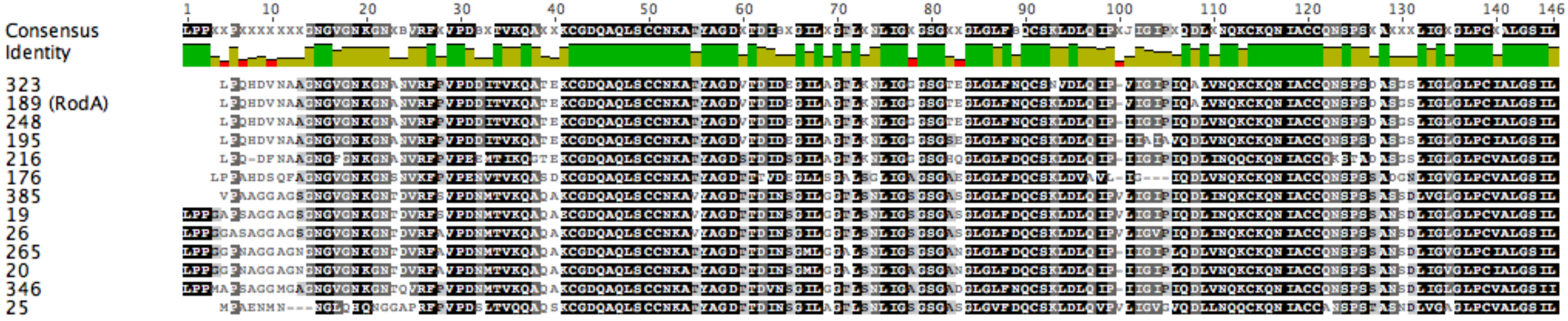

Supplementary Figure 6W. Sequence alignment and corresponding consensus sequence of proteins contained in **Region 12** (full sequence)

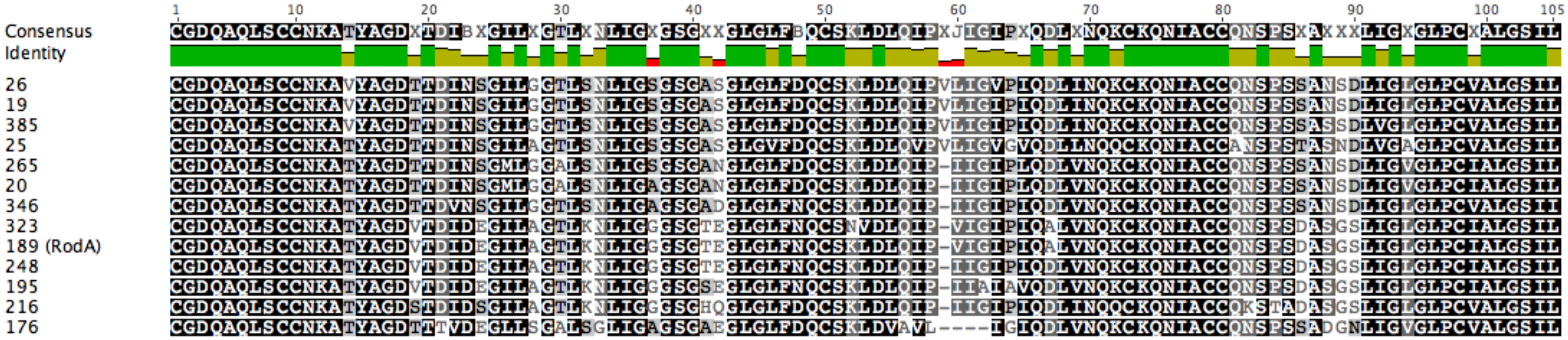

Supplementary Figure X. Sequence alignment and corresponding consensus sequence of proteins contained in **Region 12** (without N terminus region preceding first cysteine of the eight-cysteine pattern)

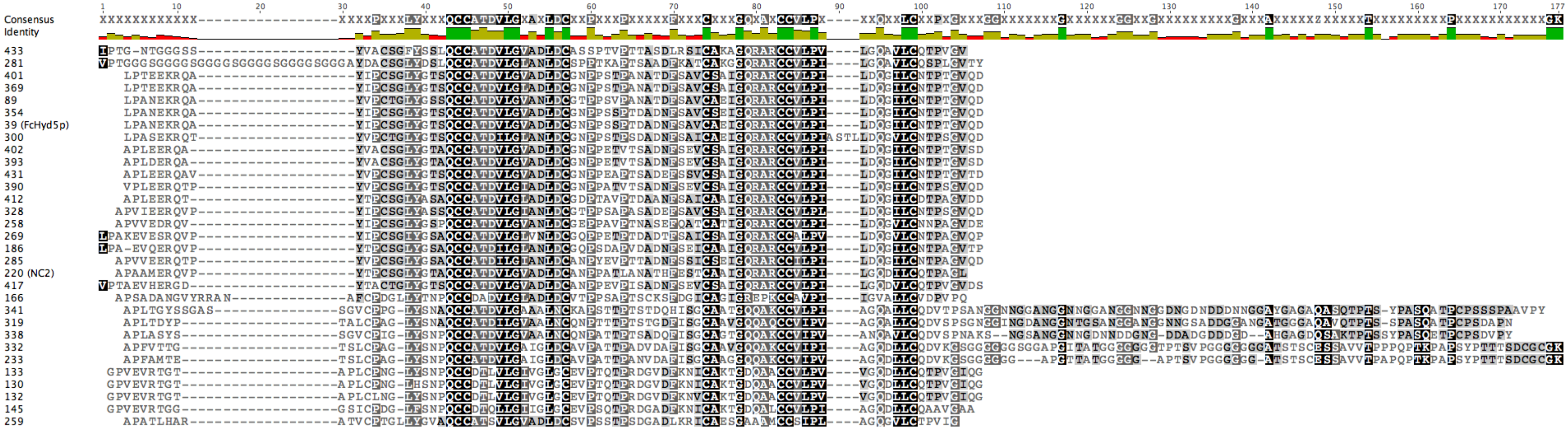

Supplementary Figure 6Y Sequence alignment and corresponding consensus sequence of proteins contained in **Region 13** (full sequence)

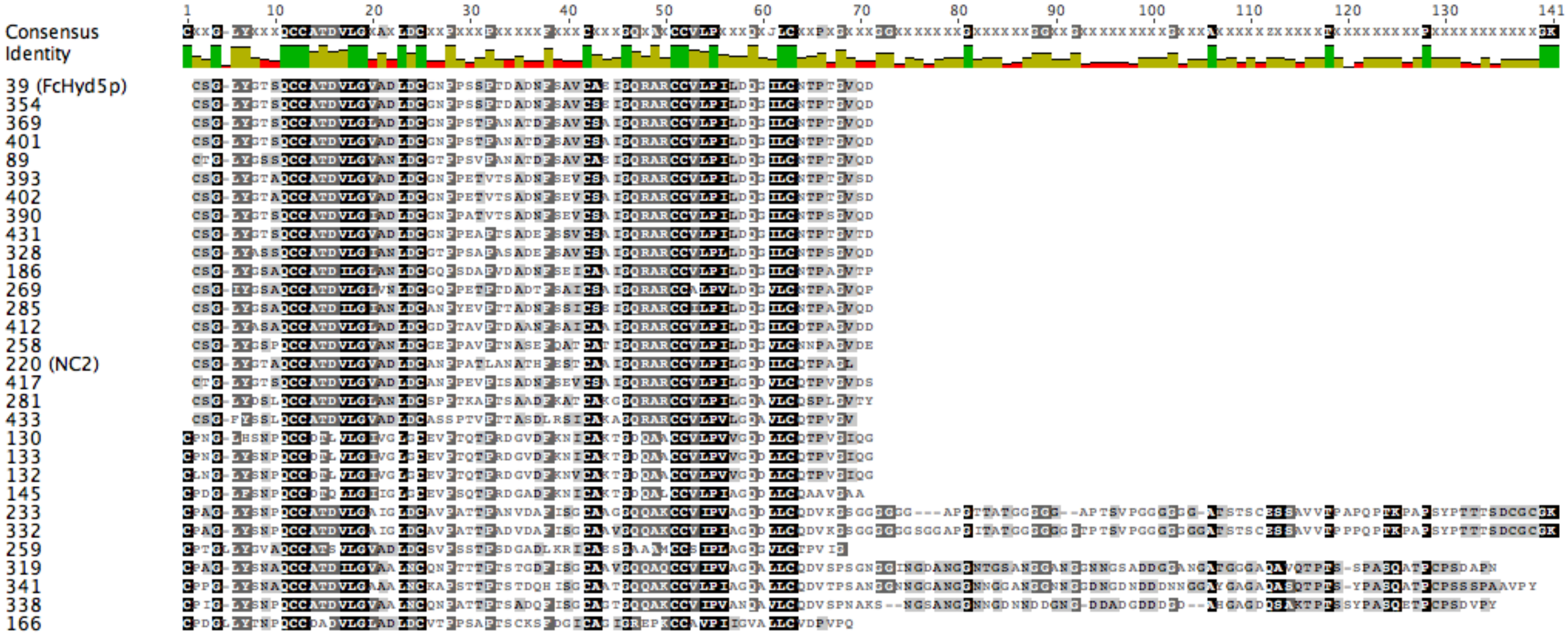

Supplementary Figure 6Z. Sequence alignment and corresponding consensus sequence of proteins contained in **Region 14** (without N terminus region preceding first cysteine of the eight-cysteine pattern)

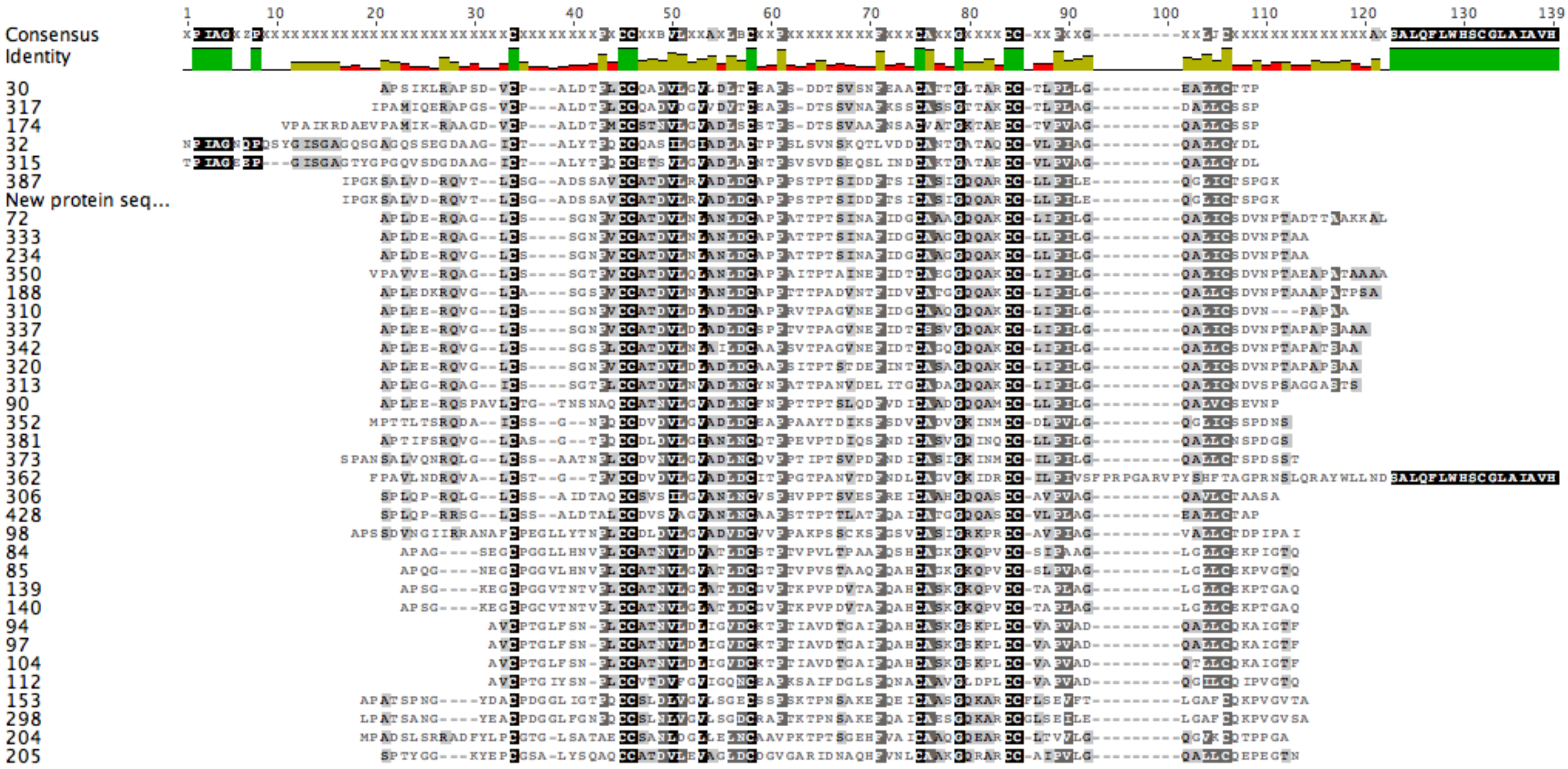

Supplementary Figure 6AA. Sequence alignment and corresponding consensus sequence of proteins contained in **Region 14** (full sequence)

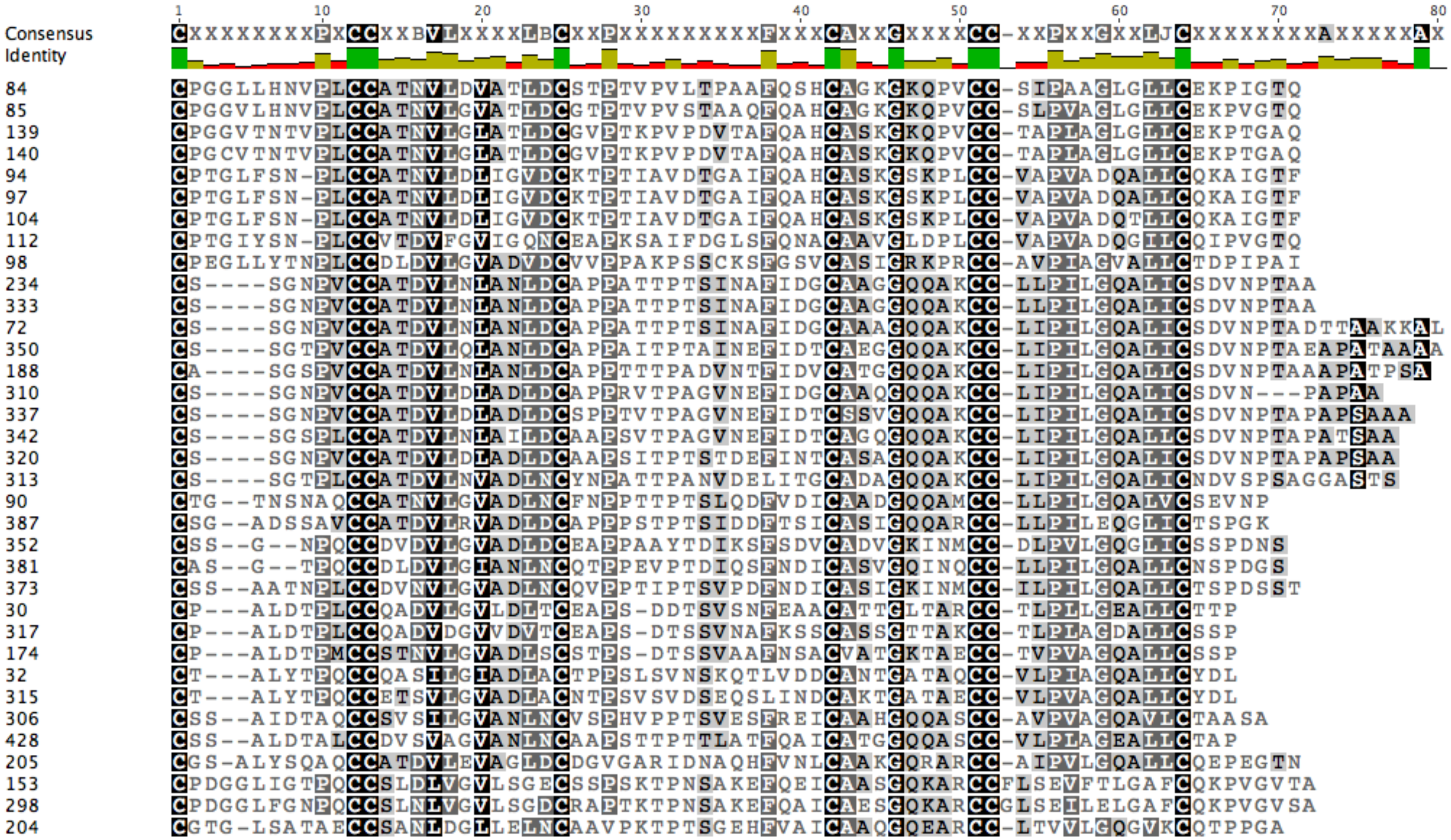

Supplementary Figure 6AB. Sequence alignment and corresponding consensus sequence of proteins contained in **Region 14** (without N terminus region preceding first cysteine of the eight-cysteine pattern)

Table 2A. Identities of region 1 (without N-terminus region preceding first cysteine of the eight-cysteine pattern)

	518	514	525	522	533	530	528	529	527	513	523	504	519	511	509	535	515	526	510	501
518		96%	98%	98%	95%	98%	98%	99%	99%	99%	99%	99%	98%	91%	91%	84%	84%	84%	73%	73%
514	96%		95%	95%	93%	95%	95%	96%	96%	96%	96%	96%	95%	89%	89%	81%	81%	81%	71%	71%
525	98%	95%		100%	98%	99%	99%	98%	98%	98%	99%	99%	98%	90%	90%	83%	83%	83%	73%	73%
522	98%	95%	100%		98%	99%	99%	98%	98%	98%	99%	99%	98%	90%	90%	83%	83%	83%	73%	73%
533	95%	93%	98%	98%		96%	96%	95%	95%	95%	96%	96%	95%	89%	89%	81%	81%	81%	71%	71%
530	98%	95%	99%	99%	96%		100%	99%	99%	99%	98%	98%	96%	90%	90%	83%	83%	83%	74%	74%
528	98%	95%	99%	99%	96%	100%		99%	99%	99%	98%	98%	96%	90%	90%	83%	83%	83%	74%	74%
529	99%	96%	98%	98%	95%	99%	99%		100%	100%	99%	99%	98%	91%	91%	84%	84%	84%	74%	74%
527	99%	96%	98%	98%	95%	99%	99%	100%		100%	99%	99%	98%	91%	91%	84%	84%	84%	74%	74%
513	99%	96%	98%	98%	95%	99%	99%	100%	100%		99%	99%	98%	91%	91%	84%	84%	84%	74%	74%
523	99%	96%	99%	99%	96%	98%	98%	99%	99%	99%		100%	99%	91%	91%	84%	84%	84%	73%	73%
504	99%	96%	99%	99%	96%	98%	98%	99%	99%	99%	100%		99%	91%	91%	84%	84%	84%	73%	73%
519	98%	95%	98%	98%	95%	96%	96%	98%	98%	98%	99%	99%		90%	90%	83%	83%	83%	73%	73%
511	91%	89%	90%	90%	89%	90%	90%	91%	91%	91%	91%	91%	90%		100%	83%	83%	83%	70%	70%
509	91%	89%	90%	90%	89%	90%	90%	91%	91%	91%	91%	91%	90%	100%		83%	83%	83%	70%	70%
535	84%	81%	83%	83%	81%	83%	83%	84%	84%	84%	84%	84%	83%	83%	83%		100%	98%	69%	69%
515	84%	81%	83%	83%	81%	83%	83%	84%	84%	84%	84%	84%	83%	83%	83%	100%		98%	69%	69%
526	84%	81%	83%	83%	81%	83%	83%	84%	84%	84%	84%	84%	83%	83%	83%	98%	98%		69%	69%
510	73%	71%	73%	73%	71%	74%	74%	74%	74%	74%	73%	73%	73%	70%	70%	69%	69%	69%		100%
501	73%	71%	73%	73%	71%	74%	74%	74%	74%	74%	73%	73%	73%	70%	70%	69%	69%	69%	100%	
750 (PcaHyd1)	71%	70%	71%	71%	69%	73%	73%	73%	73%	73%	71%	71%	71%	68%	68%	68%	68%	68%	75%	75%
965	69%	66%	69%	69%	68%	69%	69%	69%	69%	69%	69%	69%	69%	69%	69%	68%	68%	68%	66%	66%
447	77%	75%	75%	75%	74%	77%	77%	78%	78%	78%	77%	77%	77%	77%	77%	69%	69%	69%	72%	72%
443	75%	74%	74%	74%	73%	75%	75%	77%	77%	77%	75%	75%	75%	75%	75%	68%	68%	68%	69%	69%
941	74%	73%	73%	73%	72%	74%	74%	75%	75%	75%	74%	74%	74%	74%	74%	68%	68%	68%	72%	72%
454	72%	70%	70%	70%	69%	72%	72%	73%	73%	73%	72%	72%	72%	75%	75%	68%	68%	68%	69%	69%
852	72%	70%	70%	70%	68%	72%	72%	73%	73%	73%	72%	72%	72%	70%	70%	65%	65%	65%	70%	70%
448	70%	69%	69%	69%	68%	70%	70%	72%	72%	72%	70%	70%	70%	69%	69%	67%	67%	67%	73%	73%
944	77%	74%	75%	75%	74%	77%	77%	78%	78%	78%	77%	77%	75%	77%	77%	72%	72%	72%	70%	70%
622	66%	65%	66%	66%	64%	68%	68%	66%	66%	68%	66%	66%	65%	66%	66%	68%	68%	66%	63%	63%
621	64%	63%	64%	64%	61%	65%	65%	63%	63%	65%	64%	64%	63%	64%	64%	65%	65%	64%	60%	60%
1070	74%	71%	74%	74%	71%	75%	75%	73%	73%	75%	74%	74%	73%	74%	74%	69%	69%	69%	65%	65%
702	70%	69%	70%	70%	68%	71%	71%	70%	70%	71%	70%	70%	69%	69%	69%	65%	65%	64%	63%	63%
630	70%	69%	70%	70%	68%	71%	71%	70%	70%	71%	70%	70%	69%	69%	69%	68%	68%	66%	64%	64%
696	69%	68%	70%	70%	68%	69%	69%	67%	67%	69%	70%	70%	70%	69%	69%	64%	64%	64%	63%	63%
626	69%	68%	70%	70%	68%	69%	69%	67%	67%	69%	70%	70%	70%	69%	69%	64%	64%	64%	63%	63%
634	69%	68%	70%	70%	68%	69%	69%	67%	67%	69%	70%	70%	70%	69%	69%	64%	64%	64%	63%	63%
627	69%	68%	70%	70%	68%	69%	69%	67%	67%	69%	70%	70%	70%	69%	69%	64%	64%	64%	63%	63%
625	64%	63%	65%	65%	63%	64%	64%	61%	61%	64%	65%	65%	65%	64%	64%	60%	60%	60%	58%	58%

	750 (P...	965	447	443	941	454	852	448	944	622	621	1070	702	630	696	626	634	627	625
518	71%	69%	77%	75%	74%	72%	72%	70%	77%	66%	64%	74%	70%	70%	69%	69%	69%	69%	64%
514	70%	66%	75%	74%	73%	70%	70%	69%	74%	65%	63%	71%	69%	69%	68%	68%	68%	68%	63%
525	71%	69%	75%	74%	73%	70%	70%	69%	75%	66%	64%	74%	70%	70%	70%	70%	70%	70%	65%
522	71%	69%	75%	74%	73%	70%	70%	69%	75%	66%	64%	74%	70%	70%	70%	70%	70%	70%	65%
533	69%	68%	74%	73%	72%	69%	68%	68%	74%	64%	61%	71%	68%	68%	68%	68%	68%	68%	63%
530	73%	69%	77%	75%	74%	72%	72%	70%	77%	68%	65%	75%	71%	71%	69%	69%	69%	69%	64%
528	73%	69%	77%	75%	74%	72%	72%	70%	77%	68%	65%	75%	71%	71%	69%	69%	69%	69%	64%
529	73%	69%	78%	77%	75%	73%	73%	72%	78%	66%	63%	73%	70%	70%	67%	67%	67%	67%	61%
527	73%	69%	78%	77%	75%	73%	73%	72%	78%	66%	63%	73%	70%	70%	67%	67%	67%	67%	61%
513	73%	69%	78%	77%	75%	73%	73%	72%	78%	68%	65%	75%	71%	71%	69%	69%	69%	69%	64%
523	71%	69%	77%	75%	74%	72%	72%	70%	77%	66%	64%	74%	70%	70%	70%	70%	70%	70%	65%
504	71%	69%	77%	75%	74%	72%	72%	70%	77%	66%	64%	74%	70%	70%	70%	70%	70%	70%	65%
519	71%	69%	77%	75%	74%	72%	72%	70%	75%	65%	63%	73%	69%	69%	70%	70%	70%	70%	65%
511	68%	69%	77%	75%	74%	75%	70%	69%	77%	66%	64%	74%	69%	69%	69%	69%	69%	69%	64%
509	68%	69%	77%	75%	74%	75%	70%	69%	77%	66%	64%	74%	69%	69%	69%	69%	69%	69%	64%
535	68%	68%	69%	68%	68%	68%	65%	67%	72%	68%	65%	69%	65%	68%	64%	64%	64%	64%	60%
515	68%	68%	69%	68%	68%	68%	65%	67%	72%	68%	65%	69%	65%	68%	64%	64%	64%	64%	60%
526	68%	68%	69%	68%	68%	68%	65%	67%	72%	66%	64%	69%	64%	66%	64%	64%	64%	64%	60%
510	75%	66%	72%	69%	72%	69%	70%	73%	70%	63%	60%	65%	63%	64%	63%	63%	63%	63%	58%
501	75%	66%	72%	69%	72%	69%	70%	73%	70%	63%	60%	65%	63%	64%	63%	63%	63%	63%	58%
750 (PcaHyd1)		69%	72%	70%	70%	64%	74%	68%	72%	69%	68%	69%	71%	71%	71%	71%	71%	71%	66%
965	69%		67%	65%	64%	60%	73%	68%	70%	59%	56%	64%	59%	61%	63%	63%	63%	63%	58%
447	72%	67%		98%	91%	88%	83%	84%	88%	70%	68%	74%	73%	73%	72%	72%	72%	72%	68%
443	70%	65%	98%		89%	85%	81%	84%	86%	69%	67%	75%	72%	72%	69%	69%	69%	69%	65%
941	70%	64%	91%	89%		84%	81%	83%	85%	69%	67%	73%	70%	70%	69%	69%	69%	69%	65%
454	64%	60%	88%	85%	84%		79%	80%	78%	65%	63%	69%	67%	67%	67%	67%	67%	67%	63%
852	74%	73%	83%	81%	81%	79%		84%	81%	69%	67%	70%	70%	70%	69%	69%	69%	69%	64%
448	68%	68%	84%	84%	83%	80%	84%		85%	67%	64%	72%	69%	72%	68%	68%	68%	68%	63%
944	72%	70%	88%	86%	85%	78%	81%	85%		68%	65%	74%	72%	74%	68%	68%	68%	68%	63%
622	69%	59%	70%	69%	69%	65%	69%	67%	68%		98%	85%	77%	77%	76%	76%	76%	76%	73%
621	68%	56%	68%	67%	67%	63%	67%	64%	65%	98%		83%	74%	74%	76%	76%	76%	76%	73%
1070	69%	64%	74%	75%	73%	69%	70%	72%	74%	85%	83%		79%	78%	80%	79%	79%	78%	73%
702	71%	59%	73%	72%	70%	67%	70%	69%	72%	77%	74%	79%		96%	85%	84%	84%	83%	78%
630	71%	61%	73%	72%	70%	67%	70%	72%	74%	77%	74%	78%	96%		84%	84%	85%	84%	78%
696	71%	63%	72%	69%	69%	67%	69%	68%	68%	76%	76%	80%	85%	84%		99%	99%	98%	92%
626	71%	63%	72%	69%	69%	67%	69%	68%	68%	76%	76%	79%	84%	84%	99%		99%	98%	92%
634	71%	63%	72%	69%	69%	67%	69%	68%	68%	76%	76%	79%	84%	85%	99%	99%		99%	92%
627	71%	63%	72%	69%	69%	67%	69%	68%	68%	76%	76%	78%	83%	84%	98%	98%	99%		92%
625	66%	58%	68%	65%	65%	63%	64%	63%	63%	73%	73%	73%	78%	78%	92%	92%	92%	92%	

Table 2B. Identities of region 2 (without N-terminus region preceding first cysteine of the eight-cysteine pattern)

	1037	884	753	751	849	964	438	811	942	842	902	893	930
1037		56%	57%	48%	56%	51%	54%	55%	54%	57%	57%	56%	54%
884	56%		51%	45%	56%	50%	54%	56%	58%	59%	58%	53%	57%
753	57%	51%		68%	67%	55%	56%	57%	51%	57%	48%	52%	48%
751	48%	45%	68%		64%	55%	59%	60%	51%	56%	49%	54%	46%
849	56%	56%	67%	64%		64%	68%	73%	70%	70%	64%	62%	64%
964	51%	50%	55%	55%	64%		75%	78%	63%	64%	60%	63%	59%
438	54%	54%	56%	59%	68%	75%		75%	63%	68%	56%	60%	60%
811	55%	56%	57%	60%	73%	78%	75%		69%	69%	59%	63%	64%
942	54%	58%	51%	51%	70%	63%	63%	69%		73%	59%	58%	62%
842	57%	59%	57%	56%	70%	64%	68%	69%	73%		54%	52%	59%
902	57%	58%	48%	49%	64%	60%	56%	59%	59%	54%		68%	65%
893	56%	53%	52%	54%	62%	63%	60%	63%	58%	52%	68%		65%
930	54%	57%	48%	46%	64%	59%	60%	64%	62%	59%	65%	65%	
848	52%	56%	57%	54%	70%	58%	54%	67%	62%	57%	67%	60%	60%
814	51%	53%	57%	56%	70%	59%	63%	69%	59%	57%	65%	64%	59%
639	57%	59%	52%	56%	68%	63%	64%	69%	64%	60%	68%	62%	62%
933	54%	52%	54%	52%	67%	52%	52%	58%	57%	58%	58%	52%	57%
931	62%	58%	58%	54%	68%	59%	58%	62%	62%	64%	64%	59%	58%
899	60%	56%	51%	46%	67%	59%	56%	64%	63%	60%	59%	59%	64%
822	58%	49%	54%	54%	64%	56%	53%	60%	57%	63%	57%	52%	57%
815	56%	49%	51%	49%	62%	63%	62%	58%	57%	58%	46%	53%	56%
951	53%	49%	51%	47%	56%	53%	57%	57%	46%	51%	51%	47%	56%
833	59%	54%	63%	57%	64%	56%	60%	60%	56%	53%	51%	52%	59%
834	53%	46%	54%	49%	49%	46%	51%	51%	47%	48%	41%	48%	47%
821	54%	47%	63%	52%	59%	49%	54%	54%	51%	51%	46%	53%	51%
830	58%	47%	57%	49%	53%	49%	49%	52%	51%	48%	46%	49%	52%
812	54%	51%	57%	49%	60%	52%	52%	56%	51%	54%	49%	47%	54%
442	64%	56%	63%	56%	70%	54%	63%	63%	59%	62%	53%	56%	62%
828	62%	60%	60%	53%	67%	59%	65%	69%	63%	64%	53%	53%	63%

	848	814	639	933	931	899	822	815	951	833	834	821	830	812	442	828
1037	52%	51%	57%	54%	62%	60%	58%	56%	53%	59%	53%	54%	58%	54%	64%	62%
884	56%	53%	59%	52%	58%	56%	49%	49%	49%	54%	46%	47%	47%	51%	56%	60%
753	57%	57%	52%	54%	58%	51%	54%	51%	51%	63%	54%	63%	57%	57%	63%	60%
751	54%	56%	56%	52%	54%	46%	54%	49%	47%	57%	49%	52%	49%	49%	56%	53%
849	70%	70%	68%	67%	68%	67%	64%	62%	56%	64%	49%	59%	53%	60%	70%	67%
964	58%	59%	63%	52%	59%	59%	56%	63%	53%	56%	46%	49%	49%	52%	54%	59%
438	54%	63%	64%	52%	58%	56%	53%	62%	57%	60%	51%	54%	49%	52%	63%	65%
811	67%	69%	69%	58%	62%	64%	60%	58%	57%	60%	51%	54%	52%	56%	63%	69%
942	62%	59%	64%	57%	62%	63%	57%	57%	46%	56%	47%	51%	51%	51%	59%	63%
842	57%	57%	60%	58%	64%	60%	63%	58%	51%	53%	48%	51%	48%	54%	62%	64%
902	67%	65%	68%	58%	64%	59%	57%	46%	51%	51%	41%	46%	46%	49%	53%	53%
893	60%	64%	62%	52%	59%	59%	52%	53%	47%	52%	48%	53%	49%	47%	56%	53%
930	60%	59%	62%	57%	58%	64%	57%	56%	56%	59%	47%	51%	52%	54%	62%	63%
848		85%	78%	60%	60%	64%	56%	56%	54%	63%	53%	59%	53%	65%	59%	60%
814	85%		80%	59%	62%	60%	58%	56%	56%	62%	52%	59%	52%	64%	57%	64%
639	78%	80%		56%	70%	60%	59%	56%	57%	62%	56%	52%	51%	59%	58%	65%
933	60%	59%	56%		69%	67%	60%	53%	52%	57%	46%	49%	44%	51%	59%	56%
931	60%	62%	70%	69%		64%	63%	53%	54%	60%	48%	49%	48%	53%	60%	58%
899	64%	60%	60%	67%	64%		60%	60%	51%	53%	44%	53%	47%	52%	59%	58%
822	56%	58%	59%	60%	63%	60%		54%	57%	54%	51%	57%	57%	56%	60%	58%
815	56%	56%	56%	53%	53%	60%	54%		51%	56%	47%	49%	48%	56%	58%	62%
951	54%	56%	57%	52%	54%	51%	57%	51%		73%	58%	58%	63%	60%	64%	59%
833	63%	62%	62%	57%	60%	53%	54%	56%	73%		70%	72%	72%	69%	70%	65%
834	53%	52%	56%	46%	48%	44%	51%	47%	58%	70%		78%	73%	65%	56%	52%
821	59%	59%	52%	49%	49%	53%	57%	49%	58%	72%	78%		78%	70%	58%	57%
830	53%	52%	51%	44%	48%	47%	57%	48%	63%	72%	73%	78%		69%	62%	57%
812	65%	64%	59%	51%	53%	52%	56%	56%	60%	69%	65%	70%	69%		67%	59%
442	59%	57%	58%	59%	60%	59%	60%	58%	64%	70%	56%	58%	62%	67%		70%
828	60%	64%	65%	56%	58%	58%	58%	62%	59%	65%	52%	57%	57%	59%	70%	

Table 2C. Identities of region 3 (without N-terminus region preceding first cysteine of the eight-cysteine pattern)

	907	900	910	612	611	610	616	609	436	923	971	970	780	615
907		88%	73%	73%	73%	72%	65%	63%	63%	62%	52%	57%	57%	56%
900	88%		70%	70%	70%	69%	64%	64%	65%	60%	52%	57%	57%	56%
910	73%	70%		69%	69%	69%	66%	67%	65%	63%	56%	57%	54%	61%
612	73%	70%	69%		99%	98%	82%	77%	69%	69%	56%	57%	58%	64%
611	73%	70%	69%	99%		96%	82%	78%	69%	69%	56%	57%	58%	64%
610	72%	69%	69%	98%	96%		82%	75%	68%	69%	56%	57%	58%	64%
616	65%	64%	66%	82%	82%	82%		72%	62%	66%	58%	58%	55%	63%
609	63%	64%	67%	77%	78%	75%	72%		73%	62%	52%	54%	52%	64%
436	63%	65%	65%	69%	69%	68%	62%	73%		59%	60%	63%	51%	59%
923	62%	60%	63%	69%	69%	69%	66%	62%	59%		54%	55%	56%	55%
971	52%	52%	56%	56%	56%	56%	58%	52%	60%	54%		88%	63%	60%
970	57%	57%	57%	57%	57%	57%	58%	54%	63%	55%	88%		63%	56%
780	57%	57%	54%	58%	58%	58%	55%	52%	51%	56%	63%	63%		68%
615	56%	56%	61%	64%	64%	64%	63%	64%	59%	55%	60%	56%	68%	
453	63%	64%	59%	65%	65%	64%	64%	66%	69%	57%	56%	58%	53%	56%
444	64%	64%	60%	66%	66%	65%	65%	66%	69%	57%	57%	60%	54%	56%
446	64%	61%	60%	64%	64%	63%	62%	64%	66%	57%	56%	58%	57%	59%
926	67%	69%	61%	66%	66%	65%	64%	59%	61%	55%	50%	52%	53%	55%
824	58%	60%	58%	59%	59%	58%	59%	57%	52%	57%	54%	49%	52%	54%
451	54%	51%	51%	49%	49%	48%	48%	48%	49%	51%	46%	44%	51%	48%
641	55%	59%	51%	52%	52%	51%	54%	54%	52%	47%	56%	56%	52%	48%
935	59%	58%	60%	57%	58%	55%	56%	59%	58%	54%	51%	52%	58%	59%
1063	55%	57%	51%	49%	49%	48%	47%	50%	49%	50%	50%	49%	50%	49%
550	61%	59%	57%	52%	52%	51%	50%	50%	50%	50%	56%	54%	57%	52%
911	67%	68%	65%	65%	65%	63%	60%	66%	57%	59%	61%	63%	61%	64%
570	46%	49%	52%	49%	48%	49%	47%	45%	48%	44%	49%	46%	48%	49%
568	44%	41%	46%	45%	45%	45%	45%	44%	48%	46%	51%	46%	46%	45%
877	54%	56%	55%	54%	54%	52%	49%	51%	54%	52%	59%	59%	48%	52%
818	55%	57%	49%	52%	52%	54%	51%	51%	48%	55%	56%	52%	51%	49%
776	56%	56%	50%	51%	51%	51%	49%	51%	49%	51%	50%	48%	50%	48%
809	49%	48%	49%	51%	51%	51%	48%	46%	46%	54%	54%	52%	62%	54%
806	47%	42%	51%	53%	52%	53%	52%	48%	44%	53%	52%	49%	56%	54%
539 (HGFI)	52%	49%	60%	57%	57%	57%	55%	56%	49%	52%	51%	51%	57%	61%
860	58%	53%	58%	57%	57%	57%	54%	55%	55%	53%	48%	47%	52%	58%
664	48%	48%	49%	43%	43%	43%	43%	42%	46%	45%	50%	46%	47%	43%
663	48%	49%	49%	46%	46%	46%	43%	43%	48%	45%	45%	44%	48%	45%
1071	48%	51%	51%	47%	47%	46%	45%	46%	49%	45%	50%	46%	45%	44%
1059	45%	46%	46%	43%	43%	43%	39%	42%	41%	41%	46%	44%	48%	45%
1008	37%	40%	41%	36%	36%	36%	36%	35%	42%	40%	44%	43%	41%	37%
1041	53%	54%	47%	42%	42%	42%	38%	40%	45%	45%	44%	46%	49%	46%
1065	46%	46%	48%	44%	43%	44%	43%	46%	43%	45%	42%	39%	47%	48%
817	54%	51%	54%	52%	52%	52%	49%	52%	48%	52%	54%	52%	52%	53%

	453	444	446	926	824	451	641	935	1063	550	911	570	568	877
907	63%	64%	64%	67%	58%	54%	55%	59%	55%	61%	67%	46%	44%	54%
900	64%	64%	61%	69%	60%	51%	59%	58%	57%	59%	68%	49%	41%	56%
910	59%	60%	60%	61%	58%	51%	51%	60%	51%	57%	65%	52%	46%	55%
612	65%	66%	64%	66%	59%	49%	52%	57%	49%	52%	65%	49%	45%	54%
611	65%	66%	64%	66%	59%	49%	52%	58%	49%	52%	65%	48%	45%	54%
610	64%	65%	63%	65%	58%	48%	51%	55%	48%	51%	63%	49%	45%	52%
616	64%	65%	62%	64%	59%	48%	54%	56%	47%	50%	60%	47%	45%	49%
609	66%	66%	64%	59%	57%	48%	54%	59%	50%	50%	66%	45%	44%	51%
436	69%	69%	66%	61%	52%	49%	52%	58%	49%	50%	57%	48%	48%	54%
923	57%	57%	57%	55%	57%	51%	47%	54%	50%	50%	59%	44%	46%	52%
971	56%	57%	56%	50%	54%	46%	56%	51%	50%	56%	61%	49%	51%	59%
970	58%	60%	58%	52%	49%	44%	56%	52%	49%	54%	63%	46%	46%	59%
780	53%	54%	57%	53%	52%	51%	52%	58%	50%	57%	61%	48%	46%	48%
615	56%	56%	59%	55%	54%	48%	48%	59%	49%	52%	64%	49%	45%	52%
453		99%	96%	74%	68%	60%	68%	65%	45%	49%	56%	45%	39%	50%
444	99%		98%	75%	68%	62%	69%	66%	45%	49%	57%	46%	39%	50%
446	96%	98%		73%	69%	64%	67%	69%	46%	51%	60%	49%	39%	49%
926	74%	75%	73%		67%	59%	63%	65%	51%	54%	56%	45%	37%	50%
824	68%	68%	69%	67%		67%	67%	61%	51%	51%	58%	48%	42%	48%
451	60%	62%	64%	59%	67%		63%	60%	48%	51%	51%	45%	37%	40%
641	68%	69%	67%	63%	67%	63%		60%	45%	43%	55%	40%	38%	43%
935	65%	66%	69%	65%	61%	60%	60%		58%	55%	64%	50%	44%	54%
1063	45%	45%	46%	51%	51%	48%	45%	58%		68%	69%	53%	53%	58%
550	49%	49%	51%	54%	51%	51%	43%	55%	68%		67%	56%	53%	58%
911	56%	57%	60%	56%	58%	51%	55%	64%	69%	67%		57%	49%	65%
570	45%	46%	49%	45%	48%	45%	40%	50%	53%	56%	57%		65%	53%
568	39%	39%	39%	37%	42%	37%	38%	44%	53%	53%	49%	65%		54%
877	50%	50%	49%	50%	48%	40%	43%	54%	58%	58%	65%	53%	54%	
818	51%	52%	52%	57%	60%	50%	52%	54%	56%	56%	63%	52%	44%	52%
776	46%	46%	46%	50%	53%	43%	46%	55%	56%	59%	62%	52%	49%	54%
809	53%	54%	54%	49%	48%	51%	53%	54%	49%	48%	50%	46%	50%	48%
806	45%	46%	46%	48%	48%	53%	51%	54%	46%	49%	50%	44%	48%	42%
539 (HGFI)	48%	49%	49%	49%	46%	51%	48%	51%	48%	49%	62%	44%	45%	51%
860	49%	50%	50%	51%	48%	43%	44%	48%	47%	45%	52%	43%	44%	46%
664	45%	45%	44%	46%	52%	46%	44%	49%	53%	46%	50%	45%	46%	49%
663	46%	46%	45%	48%	49%	47%	42%	48%	50%	48%	46%	44%	45%	46%
1071	47%	46%	46%	47%	50%	46%	46%	47%	53%	54%	51%	45%	46%	46%
1059	38%	39%	39%	41%	42%	47%	41%	48%	48%	45%	48%	47%	47%	45%
1008	44%	42%	41%	41%	38%	44%	41%	40%	41%	39%	35%	41%	42%	38%
1041	45%	45%	45%	48%	45%	46%	44%	46%	51%	49%	50%	36%	36%	45%
1065	43%	43%	43%	48%	47%	43%	43%	48%	50%	45%	50%	44%	43%	48%
817	51%	49%	49%	51%	49%	49%	48%	53%	51%	50%	56%	44%	45%	49%

	818	776	809	806	539 (H...	860	664	663	1071	1059	1008	1041	1065	817
907	55%	56%	49%	47%	52%	58%	48%	48%	48%	45%	37%	53%	46%	54%
900	57%	56%	48%	42%	49%	53%	48%	49%	51%	46%	40%	54%	46%	51%
910	49%	50%	49%	51%	60%	58%	49%	49%	51%	46%	41%	47%	48%	54%
612	52%	51%	51%	53%	57%	57%	43%	46%	47%	43%	36%	42%	44%	52%
611	52%	51%	51%	52%	57%	57%	43%	46%	47%	43%	36%	42%	43%	52%
610	54%	51%	51%	53%	57%	57%	43%	46%	46%	43%	36%	42%	44%	52%
616	51%	49%	48%	52%	55%	54%	43%	43%	45%	39%	36%	38%	43%	49%
609	51%	51%	46%	48%	56%	55%	42%	43%	46%	42%	35%	40%	46%	52%
436	48%	49%	46%	44%	49%	55%	46%	48%	49%	41%	42%	45%	43%	48%
923	55%	51%	54%	53%	52%	53%	45%	45%	45%	41%	40%	45%	45%	52%
971	56%	50%	54%	52%	51%	48%	50%	45%	50%	46%	44%	44%	42%	54%
970	52%	48%	52%	49%	51%	47%	46%	44%	46%	44%	43%	46%	39%	52%
780	51%	50%	62%	56%	57%	52%	47%	48%	45%	48%	41%	49%	47%	52%
615	49%	48%	54%	54%	61%	58%	43%	45%	44%	45%	37%	46%	48%	53%
453	51%	46%	53%	45%	48%	49%	45%	46%	47%	38%	44%	45%	43%	51%
444	52%	46%	54%	46%	49%	50%	45%	46%	46%	39%	42%	45%	43%	49%
446	52%	46%	54%	46%	49%	50%	44%	45%	46%	39%	41%	45%	43%	49%
926	57%	50%	49%	48%	49%	51%	46%	48%	47%	41%	41%	48%	48%	51%
824	60%	53%	48%	48%	46%	48%	52%	49%	50%	42%	38%	45%	47%	49%
451	50%	43%	51%	53%	51%	43%	46%	47%	46%	47%	44%	46%	43%	49%
641	52%	46%	53%	51%	48%	44%	44%	42%	46%	41%	41%	44%	43%	48%
935	54%	55%	54%	54%	51%	48%	49%	48%	47%	48%	40%	46%	48%	53%
1063	56%	56%	49%	46%	48%	47%	53%	50%	53%	48%	41%	51%	50%	51%
550	56%	59%	48%	49%	49%	45%	46%	48%	54%	45%	39%	49%	45%	50%
911	63%	62%	50%	50%	62%	52%	50%	46%	51%	48%	35%	50%	50%	56%
570	52%	52%	46%	44%	44%	43%	45%	44%	45%	47%	41%	36%	44%	44%
568	44%	49%	50%	48%	45%	44%	46%	45%	46%	47%	42%	36%	43%	45%
877	52%	54%	48%	42%	51%	46%	49%	46%	46%	45%	38%	45%	48%	49%
818		64%	46%	50%	46%	45%	48%	48%	47%	43%	45%	48%	43%	49%
776	64%		48%	46%	43%	44%	52%	51%	51%	46%	43%	46%	49%	55%
809	46%	48%		79%	58%	53%	51%	53%	53%	54%	49%	49%	51%	53%
806	50%	46%	79%		65%	55%	48%	48%	48%	46%	45%	44%	48%	54%
539 (HGFI)	46%	43%	58%	65%		57%	48%	54%	47%	47%	41%	51%	49%	53%
860	45%	44%	53%	55%	57%		49%	55%	53%	43%	41%	48%	51%	49%
664	48%	52%	51%	48%	48%	49%		78%	61%	57%	52%	55%	53%	58%
663	48%	51%	53%	48%	54%	55%	78%		70%	55%	60%	59%	49%	49%
1071	47%	51%	53%	48%	47%	53%	61%	70%		55%	55%	52%	49%	51%
1059	43%	46%	54%	46%	47%	43%	57%	55%	55%		57%	57%	59%	52%
1008	45%	43%	49%	45%	41%	41%	52%	60%	55%	57%		52%	47%	46%
1041	48%	46%	49%	44%	51%	48%	55%	59%	52%	57%	52%		51%	52%
1065	43%	49%	51%	48%	49%	51%	53%	49%	49%	59%	47%	51%		57%
817	49%	55%	53%	54%	53%	49%	58%	49%	51%	52%	46%	52%	57%	

Table 2D. Identities of region 4 (without N-terminus region preceding first cysteine of the eight-cysteine pattern)

	1067	642	1058	631	650	647	649	648	1056	1034	629	628	635	1054	798	1064
1067		61%	63%	65%	58%	57%	52%	52%	57%	58%	60%	60%	59%	64%	65%	61%
642	61%		67%	66%	66%	62%	62%	62%	57%	55%	55%	55%	54%	59%	61%	54%
1058	63%	67%		84%	74%	78%	78%	78%	62%	67%	70%	70%	70%	73%	71%	67%
631	65%	66%	84%		68%	70%	70%	71%	66%	66%	71%	71%	71%	71%	70%	66%
650	58%	66%	74%	68%		83%	78%	79%	57%	57%	63%	63%	63%	62%	71%	66%
647	57%	62%	78%	70%	83%		78%	79%	57%	60%	60%	60%	60%	63%	70%	62%
649	52%	62%	78%	70%	78%	78%		99%	56%	54%	59%	59%	59%	59%	66%	62%
648	52%	62%	78%	71%	79%	79%	99%		57%	55%	60%	60%	60%	59%	67%	63%
1056	57%	57%	62%	66%	57%	57%	56%	57%		89%	74%	74%	74%	82%	78%	57%
1034	58%	55%	67%	66%	57%	60%	54%	55%	89%		77%	77%	76%	83%	74%	60%
629	60%	55%	70%	71%	63%	60%	59%	60%	74%	77%		100%	99%	89%	74%	72%
628	60%	55%	70%	71%	63%	60%	59%	60%	74%	77%	100%		99%	89%	74%	72%
635	59%	54%	70%	71%	63%	60%	59%	60%	74%	76%	99%	99%		88%	74%	71%
1054	64%	59%	73%	71%	62%	63%	59%	59%	82%	83%	89%	89%	88%		79%	70%
798	65%	61%	71%	70%	71%	70%	66%	67%	78%	74%	74%	74%	74%	79%		67%
1064	61%	54%	67%	66%	66%	62%	62%	63%	57%	60%	72%	72%	71%	70%	67%	
469	49%	48%	62%	60%	63%	65%	68%	70%	60%	56%	62%	62%	62%	60%	60%	60%
460	49%	48%	62%	60%	63%	65%	68%	70%	60%	56%	62%	62%	62%	60%	60%	60%
475	49%	48%	61%	60%	62%	63%	67%	68%	60%	56%	61%	61%	61%	59%	60%	59%
567	57%	56%	68%	70%	72%	70%	63%	65%	60%	60%	65%	65%	63%	62%	62%	65%
885	53%	56%	68%	63%	65%	66%	73%	73%	59%	57%	57%	57%	57%	63%	68%	59%
711	51%	54%	59%	59%	66%	66%	64%	65%	63%	61%	60%	60%	60%	61%	68%	56%
583	56%	55%	57%	55%	56%	56%	59%	59%	54%	54%	56%	56%	55%	63%	56%	56%
915	57%	52%	66%	61%	63%	66%	63%	63%	54%	54%	59%	59%	59%	63%	67%	59%
540	57%	52%	66%	61%	63%	66%	63%	63%	54%	54%	59%	59%	59%	63%	67%	59%
485	50%	48%	59%	53%	62%	60%	56%	56%	52%	53%	58%	58%	57%	59%	59%	56%
484	50%	48%	59%	53%	62%	60%	56%	56%	52%	53%	58%	58%	57%	59%	59%	56%
483	50%	48%	59%	53%	62%	60%	56%	56%	52%	53%	58%	58%	57%	59%	59%	56%
480 (SC3)	50%	48%	59%	53%	62%	60%	56%	56%	52%	53%	58%	58%	57%	59%	59%	56%
541	57%	52%	60%	59%	60%	64%	58%	58%	57%	57%	61%	61%	61%	65%	65%	60%
1060	45%	48%	53%	51%	53%	54%	52%	51%	53%	54%	57%	57%	57%	59%	57%	57%
697	44%	46%	48%	51%	52%	51%	48%	47%	52%	54%	55%	55%	55%	58%	55%	52%
1038	52%	59%	60%	55%	59%	59%	57%	57%	55%	58%	57%	57%	57%	61%	63%	59%

	469	460	475	567	885	711	583	915	540	485	484	483	480 (S...	541	1060	697	1038
1067	49%	49%	49%	57%	53%	51%	56%	57%	57%	50%	50%	50%	50%	57%	45%	44%	52%
642	48%	48%	48%	56%	56%	54%	55%	52%	52%	48%	48%	48%	48%	52%	48%	46%	59%
1058	62%	62%	61%	68%	68%	59%	57%	66%	66%	59%	59%	59%	59%	60%	53%	48%	60%
631	60%	60%	60%	70%	63%	59%	55%	61%	61%	53%	53%	53%	53%	59%	51%	51%	55%
650	63%	63%	62%	72%	65%	66%	56%	63%	63%	62%	62%	62%	62%	60%	53%	52%	59%
647	65%	65%	63%	70%	66%	66%	56%	66%	66%	60%	60%	60%	60%	64%	54%	51%	59%
649	68%	68%	67%	63%	73%	64%	59%	63%	63%	56%	56%	56%	56%	58%	52%	48%	57%
648	70%	70%	68%	65%	73%	65%	59%	63%	63%	56%	56%	56%	56%	58%	51%	47%	57%
1056	60%	60%	60%	60%	59%	63%	54%	54%	54%	52%	52%	52%	52%	57%	53%	52%	55%
1034	56%	56%	56%	60%	57%	61%	54%	54%	54%	53%	53%	53%	53%	57%	54%	54%	58%
629	62%	62%	61%	65%	57%	60%	56%	59%	59%	58%	58%	58%	58%	61%	57%	55%	57%
628	62%	62%	61%	65%	57%	60%	56%	59%	59%	58%	58%	58%	58%	61%	57%	55%	57%
635	62%	62%	61%	63%	57%	60%	55%	59%	59%	57%	57%	57%	57%	61%	57%	55%	57%
1054	60%	60%	59%	62%	63%	61%	63%	63%	63%	59%	59%	59%	59%	65%	59%	58%	61%
798	60%	60%	60%	62%	68%	68%	56%	67%	67%	59%	59%	59%	59%	65%	57%	55%	63%
1064	60%	60%	59%	65%	59%	56%	56%	59%	59%	56%	56%	56%	56%	60%	57%	52%	59%
469		100%	99%	67%	60%	63%	57%	63%	63%	62%	62%	62%	62%	60%	57%	53%	52%
460	100%		99%	67%	60%	63%	57%	63%	63%	62%	62%	62%	62%	60%	57%	53%	52%
475	99%	99%		67%	60%	63%	57%	63%	63%	60%	60%	60%	60%	59%	57%	53%	52%
567	67%	67%	67%		55%	63%	59%	65%	65%	67%	67%	67%	67%	59%	55%	53%	57%
885	60%	60%	60%	55%		75%	61%	64%	64%	57%	57%	57%	57%	61%	55%	51%	54%
711	63%	63%	63%	63%	75%		63%	62%	62%	65%	65%	65%	65%	62%	56%	52%	58%
583	57%	57%	57%	59%	61%	63%		62%	62%	56%	56%	56%	56%	58%	51%	46%	54%
915	63%	63%	63%	65%	64%	62%	62%		100%	70%	70%	70%	70%	72%	55%	52%	65%
540	63%	63%	63%	65%	64%	62%	62%	100%		70%	70%	70%	70%	72%	55%	52%	65%
485	62%	62%	60%	67%	57%	65%	56%	70%	70%		100%	100%	100%	61%	54%	50%	60%
484	62%	62%	60%	67%	57%	65%	56%	70%	70%	100%		100%	100%	61%	54%	50%	60%
483	62%	62%	60%	67%	57%	65%	56%	70%	70%	100%	100%		100%	61%	54%	50%	60%
480 (SC3)	62%	62%	60%	67%	57%	65%	56%	70%	70%	100%	100%	100%		61%	54%	50%	60%
541	60%	60%	59%	59%	61%	62%	58%	72%	72%	61%	61%	61%	61%		57%	55%	61%
1060	57%	57%	57%	55%	55%	56%	51%	55%	55%	54%	54%	54%	54%	57%		90%	63%
697	53%	53%	53%	53%	51%	52%	46%	52%	52%	50%	50%	50%	50%	55%	90%		61%
1038	52%	52%	52%	57%	54%	58%	54%	65%	65%	60%	60%	60%	60%	61%	63%	61%	

Table 2E. Identities of region 5 (without N-terminus region preceding first cysteine of the eight-cysteine pattern)

	1068	698	700	1046	1040	1049	1048	1053	1050	1047	1055	1051	1061	589	587	999	575	557	555	918	1066	1000 (...	477	471	459	476	468	458	586	920	921	710	552	643	916
1068		89%	79%	84%	85%	87%	82%	72%	77%	73%	52%	54%	54%	46%	50%	49%	52%	48%	51%	53%	66%	48%	48%	49%	49%	49%	54%	51%	56%	54%	48%	48%	50%	63%	51%
698	89%		80%	84%	83%	84%	77%	72%	78%	78%	56%	57%	57%	49%	52%	49%	52%	48%	51%	57%	68%	53%	53%	54%	54%	54%	57%	52%	59%	55%	52%	52%	52%	59%	57%
700	79%	80%		74%	73%	79%	70%	72%	75%	74%	49%	51%	50%	44%	44%	45%	46%	45%	44%	53%	62%	46%	46%	48%	48%	49%	51%	49%	54%	48%	45%	48%	46%	55%	45%
1046	84%	84%	74%		99%	88%	77%	77%	76%	73%	60%	60%	57%	54%	54%	51%	51%	47%	56%	53%	67%	50%	50%	51%	51%	50%	54%	49%	56%	54%	53%	54%	52%	60%	52%
1040	85%	83%	73%	99%		89%	78%	76%	75%	72%	59%	59%	57%	54%	54%	51%	52%	47%	58%	53%	67%	50%	50%	51%	51%	50%	54%	49%	56%	54%	52%	53%	51%	60%	51%
1049	87%	84%	79%	88%	89%		79%	75%	77%	76%	54%	56%	54%	49%	50%	51%	53%	49%	53%	56%	66%	51%	51%	52%	52%	52%	54%	49%	56%	54%	49%	51%	52%	61%	48%
1048	82%	77%	70%	77%	78%	79%		65%	66%	67%	51%	55%	54%	49%	52%	48%	46%	44%	48%	51%	62%	46%	46%	48%	48%	49%	54%	52%	59%	54%	51%	48%	44%	59%	48%
1053	72%	72%	72%	77%	76%	75%	65%		89%	82%	59%	57%	55%	53%	55%	48%	48%	45%	51%	51%	61%	45%	45%	46%	46%	43%	51%	47%	55%	51%	49%	49%	51%	61%	53%
1050	77%	78%	75%	76%	75%	77%	66%	89%		88%	55%	53%	54%	51%	52%	51%	50%	47%	50%	54%	64%	48%	48%	49%	49%	48%	54%	49%	58%	52%	52%	49%	52%	65%	57%
1047	73%	78%	74%	73%	72%	76%	67%	82%	88%		56%	52%	54%	50%	51%	50%	49%	47%	47%	54%	61%	48%	48%	49%	49%	49%	52%	49%	57%	53%	53%	49%	49%	59%	57%
1055	52%	56%	49%	60%	59%	54%	51%	59%	55%	56%		74%	65%	63%	59%	57%	54%	52%	61%	51%	63%	51%	51%	52%	52%	59%	63%	61%	61%	47%	57%	58%	56%	52%	54%
1051	54%	57%	51%	60%	59%	56%	55%	57%	53%	52%	74%		68%	63%	60%	61%	60%	55%	66%	52%	61%	52%	52%	54%	54%	55%	59%	54%	55%	47%	55%	52%	51%	48%	55%
1061	54%	57%	50%	57%	57%	54%	54%	55%	54%	54%	65%	68%		60%	62%	56%	53%	48%	59%	57%	59%	49%	49%	50%	50%	56%	59%	56%	57%	47%	49%	48%	46%	51%	45%
589	46%	49%	44%	54%	54%	49%	49%	53%	51%	50%	63%	63%	60%		68%	67%	62%	59%	64%	58%	57%	57%	57%	59%	59%	63%	65%	66%	66%	58%	57%	61%	54%	54%	43%
587	50%	52%	44%	54%	54%	50%	52%	55%	52%	51%	59%	60%	62%	68%		62%	53%	49%	58%	59%	56%	52%	52%	54%	54%	60%	56%	55%	61%	52%	52%	54%	46%	51%	41%
999	49%	49%	45%	51%	51%	51%	48%	48%	51%	50%	57%	61%	56%	67%	62%		66%	60%	60%	57%	60%	54%	54%	55%	55%	62%	62%	62%	62%	54%	49%	59%	55%	53%	40%
575	52%	52%	46%	51%	52%	53%	46%	48%	50%	49%	54%	60%	53%	62%	53%	66%		82%	74%	55%	60%	64%	64%	65%	65%	61%	62%	61%	59%	57%	49%	53%	59%	51%	47%
557	48%	48%	45%	47%	47%	49%	44%	45%	47%	47%	52%	55%	48%	59%	49%	60%	82%		67%	49%	55%	54%	54%	55%	55%	56%	55%	58%	53%	55%	48%	49%	55%	47%	40%
555	51%	51%	44%	56%	58%	53%	48%	51%	50%	47%	61%	66%	59%	64%	58%	60%	74%	67%		51%	58%	56%	56%	58%	58%	61%	60%	58%	56%	56%	49%	52%	53%	49%	46%
918	53%	57%	53%	53%	53%	56%	51%	51%	54%	54%	51%	52%	57%	58%	59%	57%	55%	49%	51%		60%	56%	56%	57%	57%	60%	59%	58%	58%	57%	52%	52%	51%	54%	42%
1066	66%	68%	62%	67%	67%	66%	62%	61%	64%	61%	63%	61%	59%	57%	56%	60%	60%	55%	58%	60%		62%	62%	63%	63%	61%	71%	66%	72%	58%	55%	59%	55%	64%	44%
1000 (VMH2)	48%	53%	46%	50%	50%	51%	46%	45%	48%	48%	51%	52%	49%	57%	52%	54%	64%	54%	56%	56%	62%		100%	99%	99%	82%	73%	74%	67%	61%	51%	54%	60%	53%	48%
477	48%	53%	46%	50%	50%	51%	46%	45%	48%	48%	51%	52%	49%	57%	52%	54%	64%	54%	56%	56%	62%	100%		99%	99%	82%	73%	74%	67%	61%	51%	54%	60%	53%	48%
471	49%	54%	48%	51%	51%	52%	48%	46%	49%	49%	52%	54%	50%	59%	54%	55%	65%	55%	58%	57%	63%	99%	99%		100%	83%	74%	76%	68%	63%	52%	55%	61%	54%	49%
459	49%	54%	48%	51%	51%	52%	48%	46%	49%	49%	52%	54%	50%	59%	54%	55%	65%	55%	58%	57%	63%	99%	99%	100%		83%	74%	76%	68%	63%	52%	55%	61%	54%	49%
476	49%	54%	49%	50%	50%	52%	49%	43%	48%	49%	59%	55%	56%	63%	60%	62%	61%	56%	61%	60%	61%	82%	82%	83%	83%		73%	76%	67%	64%	53%	57%	60%	51%	44%
468	54%	57%	51%	54%	54%	54%	54%	51%	54%	52%	63%	59%	59%	65%	56%	62%	62%	55%	60%	59%	71%	73%	73%	74%	74%	73%		89%	89%	65%	49%	60%	60%	58%	46%
458	51%	52%	49%	49%	49%	49%	52%	47%	49%	49%	61%	54%	56%	66%	55%	62%	61%	58%	58%	58%	66%	74%	74%	76%	76%	76%	89%		84%	64%	51%	61%	61%	60%	44%
586	56%	59%	54%	56%	56%	56%	59%	55%	58%	57%	61%	55%	57%	66%	61%	62%	59%	53%	56%	58%	72%	67%	67%	68%	68%	67%	89%	84%		67%	53%	63%	59%	64%	49%
920	54%	55%	48%	54%	54%	54%	54%	51%	52%	53%	47%	47%	47%	58%	52%	54%	57%	55%	56%	57%	58%	61%	61%	63%	63%	64%	65%	64%	67%		55%	58%	53%	50%	49%
921	48%	52%	45%	53%	52%	49%	51%	49%	52%	53%	57%	55%	49%	57%	52%	49%	49%	48%	49%	52%	55%	51%	51%	52%	52%	53%	49%	51%	53%	55%		55%	42%	43%	49%
710	48%	52%	48%	54%	53%	51%	48%	49%	49%	49%	58%	52%	48%	61%	54%	59%	53%	49%	52%	52%	59%	54%	54%	55%	55%	57%	60%	61%	63%	58%	55%		57%	54%	46%
552	50%	52%	46%	52%	51%	52%	44%	51%	52%	49%	56%	51%	46%	54%	46%	55%	59%	55%	53%	51%	55%	60%	60%	61%	61%	60%	60%	61%	59%	53%	42%	57%		57%	43%
643	63%	59%	55%	60%	60%	61%	59%	61%	65%	59%	52%	48%	51%	54%	51%	53%	51%	47%	49%	54%	64%	53%	53%	54%	54%	51%	58%	60%	64%	50%	43%	54%	57%		46%
916	51%	57%	45%	52%	51%	48%	48%	53%	57%	57%	54%	55%	45%	43%	41%	40%	47%	40%	46%	42%	44%	48%	48%	49%	49%	44%	46%	44%	49%	49%	49%	46%	43%	46%	

Table 2F. Identities region 6 (without N-terminus region preceding first cysteine of the eight-cysteine pattern)

	990	489	737	988	738	729 (S…	728	987	974	986	733	629	659	495	493	670	770	763	768	758	716	715	608	581	876	546	661	657	956
990		99%	79%	39%	39%	38%	39%	43%	39%	37%	37%	39%	33%	33%	33%	29%	33%	33%	34%	36%	36%	33%	32%	32%	38%	38%	37%	33%	27%
489	99%		78%	39%	39%	38%	39%	43%	39%	37%	37%	39%	33%	33%	33%	29%	31%	31%	33%	35%	36%	33%	32%	32%	38%	37%	37%	33%	27%
737	79%	78%		40%	39%	39%	39%	40%	39%	43%	41%	38%	35%	35%	35%	35%	33%	33%	34%	35%	34%	32%	30%	32%	38%	38%	39%	35%	25%
988	39%	39%	40%		81%	49%	51%	47%	42%	51%	46%	46%	36%	36%	36%	38%	31%	31%	32%	41%	34%	33%	40%	38%	39%	36%	36%	42%	32%
738	39%	39%	39%	81%		46%	47%	43%	40%	46%	44%	43%	33%	33%	33%	33%	32%	32%	31%	39%	33%	34%	41%	35%	37%	35%	35%	38%	30%
729 (SC16)	38%	38%	39%	49%	46%		96%	41%	40%	44%	41%	41%	36%	36%	36%	41%	30%	30%	30%	38%	34%	33%	34%	36%	40%	33%	42%	41%	27%
728	39%	39%	39%	51%	47%	96%		41%	40%	44%	41%	43%	36%	36%	36%	40%	31%	31%	31%	39%	33%	34%	35%	36%	40%	34%	41%	40%	28%
987	43%	43%	40%	47%	43%	41%	41%		83%	75%	78%	51%	36%	36%	36%	45%	36%	36%	38%	39%	40%	41%	43%	41%	46%	41%	36%	42%	33%
974	39%	39%	39%	42%	40%	40%	40%	83%		74%	78%	48%	33%	33%	33%	36%	34%	34%	35%	35%	36%	36%	45%	41%	43%	41%	39%	45%	34%
986	37%	37%	43%	51%	46%	44%	44%	75%	74%		83%	46%	37%	37%	37%	40%	32%	32%	33%	41%	42%	37%	46%	43%	41%	40%	40%	45%	31%
733	37%	37%	41%	46%	44%	41%	41%	78%	78%	83%		46%	39%	39%	39%	40%	38%	38%	39%	44%	40%	36%	48%	42%	42%	42%	36%	40%	29%
629	39%	39%	38%	46%	43%	41%	43%	51%	48%	46%	46%		38%	38%	38%	39%	34%	34%	35%	40%	38%	43%	45%	45%	43%	44%	43%	46%	33%
659	33%	33%	35%	36%	33%	36%	36%	36%	33%	37%	39%	38%		100%	100%	40%	28%	28%	28%	42%	38%	33%	41%	45%	42%	34%	37%	39%	26%
495	33%	33%	35%	36%	33%	36%	36%	36%	33%	37%	39%	38%	100%		100%	40%	28%	28%	28%	42%	38%	33%	41%	45%	42%	34%	37%	39%	26%
493	33%	33%	35%	36%	33%	36%	36%	36%	33%	37%	39%	38%	100%	100%		40%	28%	28%	28%	42%	38%	33%	41%	45%	42%	34%	37%	39%	26%
670	29%	29%	35%	38%	33%	41%	40%	45%	36%	40%	40%	39%	40%	40%	40%		31%	31%	31%	32%	33%	37%	42%	39%	36%	36%	36%	40%	33%
770	33%	31%	33%	31%	32%	30%	31%	36%	34%	32%	38%	34%	28%	28%	28%	31%		100%	98%	48%	37%	37%	43%	40%	36%	33%	36%	34%	26%
763	33%	31%	33%	31%	32%	30%	31%	36%	34%	32%	38%	34%	28%	28%	28%	31%	100%		98%	48%	37%	37%	43%	40%	36%	33%	36%	34%	26%
768	34%	33%	34%	32%	31%	30%	31%	38%	35%	33%	39%	35%	28%	28%	28%	31%	98%	98%		48%	36%	36%	43%	40%	36%	34%	38%	35%	26%
758	36%	35%	35%	41%	39%	38%	39%	39%	35%	41%	44%	40%	42%	42%	42%	32%	48%	48%	48%		45%	45%	47%	47%	41%	39%	35%	38%	30%
716	36%	36%	34%	34%	33%	34%	33%	40%	36%	42%	40%	38%	38%	38%	38%	33%	37%	37%	36%	45%		56%	47%	44%	42%	38%	36%	38%	31%
715	33%	33%	32%	33%	34%	33%	34%	41%	36%	37%	36%	43%	33%	33%	33%	37%	37%	37%	36%	45%	56%		43%	38%	38%	41%	33%	38%	30%
608	32%	32%	30%	40%	41%	34%	35%	43%	45%	46%	48%	45%	41%	41%	41%	42%	43%	43%	43%	47%	47%	43%		62%	42%	47%	45%	48%	27%
581	32%	32%	32%	38%	35%	36%	36%	41%	41%	43%	42%	45%	45%	45%	45%	39%	40%	40%	40%	47%	44%	38%	62%		45%	45%	52%	43%	33%
876	38%	38%	38%	39%	37%	40%	40%	46%	43%	41%	42%	43%	42%	42%	42%	36%	36%	36%	36%	41%	42%	38%	42%	45%		48%	46%	45%	36%
546	38%	37%	38%	36%	35%	33%	34%	41%	41%	40%	42%	44%	34%	34%	34%	36%	33%	33%	34%	39%	38%	41%	47%	45%	48%		47%	45%	29%
661	37%	37%	39%	36%	35%	42%	41%	36%	39%	40%	36%	43%	37%	37%	37%	36%	36%	36%	38%	35%	36%	33%	45%	52%	46%	47%		69%	36%
657	33%	33%	35%	42%	38%	41%	40%	42%	45%	45%	40%	46%	39%	39%	39%	40%	34%	34%	35%	38%	38%	38%	48%	43%	45%	45%	69%		39%
956	27%	27%	25%	32%	30%	27%	28%	33%	34%	31%	29%	33%	26%	26%	26%	33%	26%	26%	26%	30%	31%	30%	27%	33%	36%	29%	36%	39%	

Table 2G. Identities region 7 (without N-terminus region preceding first cysteine of the eight-cysteine pattern)

	1062	1043	1004	1003	841	839	840	837	423	242	998	983	742	709	675	996	975	966	959	908	892	927
1062		58%	31%	29%	26%	24%	23%	23%	29%	22%	21%	24%	23%	32%	25%	28%	33%	19%	16%	23%	22%	26%
1043	58%		26%	29%	24%	24%	22%	22%	26%	23%	23%	25%	24%	27%	27%	24%	25%	17%	16%	24%	27%	23%
1004	31%	26%		59%	25%	22%	21%	27%	24%	25%	23%	22%	22%	23%	22%	28%	27%	17%	18%	20%	20%	19%
1003	29%	29%	59%		24%	23%	22%	34%	21%	27%	20%	25%	25%	23%	23%	24%	27%	20%	20%	22%	21%	20%
841	26%	24%	25%	24%		83%	68%	54%	24%	21%	22%	23%	23%	29%	20%	31%	27%	22%	19%	19%	24%	20%
839	24%	24%	22%	23%	83%		64%	53%	25%	23%	22%	22%	22%	25%	21%	27%	25%	14%	16%	16%	21%	22%
840	23%	22%	21%	22%	68%	64%		48%	19%	22%	16%	24%	24%	29%	23%	30%	24%	21%	18%	18%	24%	19%
837	23%	22%	27%	34%	54%	53%	48%		20%	23%	21%	24%	24%	27%	24%	29%	26%	17%	17%	16%	22%	23%
423	29%	26%	24%	21%	24%	25%	19%	20%		38%	34%	26%	26%	27%	23%	33%	27%	22%	17%	26%	17%	26%
242	22%	23%	25%	27%	21%	23%	22%	23%	38%		28%	26%	26%	23%	26%	23%	22%	20%	20%	16%	23%	21%
998	21%	23%	23%	20%	22%	22%	16%	21%	34%	28%		22%	22%	27%	20%	22%	25%	15%	20%	24%	16%	21%
983	24%	25%	22%	25%	23%	22%	24%	24%	26%	26%	22%		99%	34%	36%	24%	25%	21%	21%	28%	28%	20%
742	23%	24%	22%	25%	23%	22%	24%	24%	26%	26%	22%	99%		33%	35%	24%	25%	21%	21%	28%	27%	20%
709	32%	27%	23%	23%	29%	25%	29%	27%	27%	23%	27%	34%	33%		42%	31%	32%	32%	24%	29%	27%	26%
675	25%	27%	22%	23%	20%	21%	23%	24%	23%	26%	20%	36%	35%	42%		23%	26%	21%	19%	25%	26%	29%
996	28%	24%	28%	24%	31%	27%	30%	29%	33%	23%	22%	24%	24%	31%	23%		39%	23%	22%	29%	26%	28%
975	33%	25%	27%	27%	27%	25%	24%	26%	27%	22%	25%	25%	25%	32%	26%	39%		25%	19%	25%	19%	26%
966	19%	17%	17%	20%	22%	14%	21%	17%	22%	20%	15%	21%	21%	32%	21%	23%	25%		59%	29%	33%	16%
959	16%	16%	18%	20%	19%	16%	18%	17%	17%	20%	20%	21%	21%	24%	19%	22%	19%	59%		27%	31%	16%
908	23%	24%	20%	22%	19%	16%	18%	16%	26%	16%	24%	28%	28%	29%	25%	29%	25%	29%	27%		38%	26%
892	22%	27%	20%	21%	24%	21%	24%	22%	17%	23%	16%	28%	27%	27%	26%	26%	19%	33%	31%	38%		19%
927	26%	23%	19%	20%	20%	22%	19%	23%	26%	21%	21%	20%	20%	26%	29%	28%	26%	16%	16%	26%	19%	

Table 2H. Identities region 8 (without N-terminus region preceding first cysteine of the eight-cysteine pattern)

	425	421	260	24	384	178	937	380	193	88	40	226	38	31	37	36	35	329	286	432	219 (E...	389	372	429	803	18	321
425		70%	68%	69%	65%	70%	32%	20%	28%	13%	14%	18%	19%	19%	19%	19%	20%	15%	19%	15%	16%	17%	19%	10%	23%	16%	8%
421	70%		61%	63%	63%	64%	36%	23%	28%	14%	14%	17%	14%	14%	14%	14%	17%	18%	19%	16%	19%	19%	20%	11%	22%	16%	9%
260	68%	61%		95%	92%	74%	32%	23%	30%	14%	15%	18%	18%	18%	19%	19%	19%	17%	20%	15%	20%	18%	19%	13%	19%	17%	11%
24	69%	63%	95%		92%	72%	33%	24%	29%	14%	15%	18%	18%	18%	19%	19%	19%	17%	20%	15%	20%	18%	19%	12%	19%	17%	11%
384	65%	63%	92%	92%		73%	34%	23%	31%	13%	14%	16%	18%	18%	19%	19%	19%	17%	20%	15%	20%	18%	19%	12%	20%	17%	9%
178	70%	64%	74%	72%	73%		33%	21%	28%	13%	14%	19%	16%	16%	18%	18%	18%	17%	22%	16%	20%	16%	19%	14%	22%	19%	7%
937	32%	36%	32%	33%	34%	33%		24%	28%	15%	15%	13%	20%	20%	17%	17%	20%	20%	23%	21%	14%	19%	13%	15%	22%	19%	11%
380	20%	23%	23%	24%	23%	21%	24%		34%	28%	23%	23%	20%	20%	18%	18%	20%	13%	18%	14%	26%	15%	19%	18%	18%	22%	12%
193	28%	28%	30%	29%	31%	28%	28%	34%		18%	26%	20%	17%	17%	20%	20%	17%	14%	16%	13%	25%	14%	16%	19%	23%	23%	9%
88	13%	14%	14%	14%	13%	13%	15%	28%	18%		55%	47%	16%	18%	18%	19%	16%	14%	14%	15%	24%	10%	14%	17%	20%	27%	9%
40	14%	14%	15%	15%	14%	14%	15%	23%	26%	55%		45%	12%	14%	14%	14%	15%	15%	14%	17%	23%	11%	16%	13%	16%	25%	8%
226	18%	17%	18%	18%	16%	19%	13%	23%	20%	47%	45%		17%	18%	17%	17%	15%	15%	14%	18%	23%	13%	12%	18%	17%	27%	8%
38	19%	14%	18%	18%	18%	16%	20%	20%	17%	16%	12%	17%		97%	77%	79%	77%	14%	13%	20%	17%	15%	12%	24%	17%	21%	7%
31	19%	14%	18%	18%	18%	16%	20%	20%	17%	18%	14%	18%	97%		80%	81%	80%	14%	13%	20%	18%	15%	12%	23%	18%	21%	7%
37	19%	14%	19%	19%	19%	18%	17%	18%	20%	18%	14%	17%	77%	80%		99%	73%	12%	12%	19%	20%	14%	14%	23%	18%	18%	7%
36	19%	14%	19%	19%	19%	18%	17%	18%	20%	19%	14%	17%	79%	81%	99%		73%	12%	12%	19%	20%	14%	14%	23%	18%	17%	7%
35	20%	17%	19%	19%	19%	18%	20%	20%	17%	16%	15%	15%	77%	80%	73%	73%		14%	13%	19%	18%	15%	12%	25%	18%	20%	6%
329	15%	18%	17%	17%	17%	17%	20%	13%	14%	14%	15%	15%	14%	14%	12%	12%	14%		56%	45%	14%	28%	27%	11%	16%	13%	11%
286	19%	19%	20%	20%	20%	22%	23%	18%	16%	14%	14%	14%	13%	13%	12%	12%	13%	56%		42%	14%	29%	33%	12%	16%	14%	8%
432	15%	16%	15%	15%	15%	16%	21%	14%	13%	15%	17%	18%	20%	20%	19%	19%	19%	45%	42%		16%	25%	22%	12%	14%	17%	8%
219 (EAS)	16%	19%	20%	20%	20%	20%	14%	26%	25%	24%	23%	23%	17%	18%	20%	20%	18%	14%	14%	16%		17%	19%	20%	17%	20%	10%
389	17%	19%	18%	18%	18%	16%	19%	15%	14%	10%	11%	13%	15%	15%	14%	14%	15%	28%	29%	25%	17%		26%	13%	16%	16%	12%
372	19%	20%	19%	19%	19%	19%	13%	19%	16%	14%	16%	12%	12%	12%	14%	14%	12%	27%	33%	22%	19%	26%		9%	8%	11%	11%
429	10%	11%	13%	12%	12%	14%	15%	18%	19%	17%	13%	18%	24%	23%	23%	23%	25%	11%	12%	12%	20%	13%	9%		17%	19%	8%
803	23%	22%	19%	19%	20%	22%	22%	18%	23%	20%	16%	17%	17%	18%	18%	18%	18%	16%	16%	14%	17%	16%	8%	17%		20%	9%
18	16%	16%	17%	17%	17%	19%	19%	22%	23%	27%	25%	27%	21%	21%	18%	17%	20%	13%	14%	17%	20%	16%	11%	19%	20%		7%
321	8%	9%	11%	11%	9%	7%	11%	12%	9%	9%	8%	8%	7%	7%	7%	7%	6%	11%	8%	8%	10%	12%	11%	8%	9%	7%	

Table 2I. Identities region 9 (without N-terminus region preceding first cysteine of the eight-cysteine pattern)

	866	861	854	853	424	592	358	229	177 (D…	165	262	261	21	356	224	377	183 (M…	225	973	380	294	330	207	34	427	304
866		83%	76%	39%	26%	35%	18%	19%	14%	14%	11%	12%	12%	16%	16%	13%	17%	16%	25%	24%	20%	16%	16%	14%	12%	19%
861	83%		73%	39%	25%	30%	16%	18%	13%	13%	11%	13%	13%	16%	16%	11%	15%	18%	25%	25%	19%	19%	17%	16%	13%	20%
854	76%	73%		39%	26%	31%	18%	18%	12%	12%	11%	12%	12%	17%	18%	11%	17%	15%	25%	21%	20%	19%	19%	17%	15%	19%
853	39%	39%	39%		36%	28%	16%	16%	12%	12%	12%	11%	11%	13%	13%	10%	13%	15%	32%	20%	15%	17%	17%	17%	13%	15%
424	26%	25%	26%	36%		27%	15%	16%	17%	17%	14%	15%	15%	15%	15%	9%	13%	12%	27%	21%	16%	17%	18%	14%	13%	14%
592	35%	30%	31%	28%	27%		15%	15%	18%	18%	16%	14%	14%	19%	18%	10%	15%	15%	31%	22%	19%	17%	16%	15%	11%	18%
358	18%	16%	18%	16%	15%	15%		94%	41%	41%	45%	31%	31%	19%	19%	13%	16%	12%	15%	13%	14%	12%	10%	10%	15%	8%
229	19%	18%	18%	16%	16%	15%	94%		43%	43%	47%	31%	31%	19%	19%	13%	16%	12%	15%	15%	15%	12%	11%	10%	14%	9%
177 (DewA)	14%	13%	12%	12%	17%	18%	41%	43%		100%	60%	39%	39%	22%	20%	13%	15%	12%	13%	16%	10%	17%	11%	11%	13%	10%
165	14%	13%	12%	12%	17%	18%	41%	43%	100%		60%	39%	39%	22%	20%	13%	15%	12%	13%	16%	10%	17%	11%	11%	13%	10%
262	11%	11%	11%	12%	14%	16%	45%	47%	60%	60%		49%	49%	16%	16%	14%	17%	13%	15%	13%	11%	16%	14%	10%	9%	11%
261	12%	13%	12%	11%	15%	14%	31%	31%	39%	39%	49%		99%	16%	18%	12%	15%	10%	11%	10%	8%	12%	14%	9%	11%	9%
21	12%	13%	12%	11%	15%	14%	31%	31%	39%	39%	49%	99%		16%	18%	12%	15%	10%	11%	10%	8%	12%	14%	9%	11%	9%
356	16%	16%	17%	13%	15%	19%	19%	19%	22%	22%	16%	16%	16%		92%	12%	15%	13%	14%	17%	18%	16%	10%	14%	9%	13%
224	16%	16%	18%	13%	15%	18%	19%	19%	20%	20%	16%	18%	18%	92%		12%	15%	13%	14%	15%	18%	15%	11%	14%	9%	13%
377	13%	11%	11%	10%	9%	10%	13%	13%	13%	13%	14%	12%	12%	12%	12%		69%	17%	8%	11%	12%	14%	10%	10%	5%	8%
183 (MPG1)	17%	15%	17%	13%	13%	15%	16%	16%	15%	15%	17%	15%	15%	15%	15%	69%		26%	11%	16%	15%	17%	13%	12%	7%	11%
225	16%	18%	15%	15%	12%	15%	12%	12%	12%	12%	13%	10%	10%	13%	13%	17%	26%		14%	15%	13%	13%	12%	15%	15%	12%
973	25%	25%	25%	32%	27%	31%	15%	15%	13%	13%	15%	11%	11%	14%	14%	8%	11%	14%		26%	22%	19%	14%	15%	14%	16%
380	24%	25%	21%	20%	21%	22%	13%	15%	16%	16%	13%	10%	10%	17%	15%	11%	16%	15%	26%		18%	21%	14%	17%	13%	13%
294	20%	19%	20%	15%	16%	19%	14%	15%	10%	10%	11%	8%	8%	18%	18%	12%	15%	13%	22%	18%		13%	17%	12%	12%	16%
330	16%	19%	19%	17%	17%	17%	12%	12%	17%	17%	16%	12%	12%	16%	15%	14%	17%	13%	19%	21%	13%		17%	13%	11%	15%
207	16%	17%	19%	17%	18%	16%	10%	11%	11%	11%	14%	14%	14%	10%	11%	10%	13%	12%	14%	14%	17%	17%		11%	11%	13%
34	14%	16%	17%	17%	14%	15%	10%	10%	11%	11%	10%	9%	9%	14%	14%	10%	12%	15%	15%	17%	12%	13%	11%		31%	19%
427	12%	13%	15%	13%	13%	11%	15%	14%	13%	13%	9%	11%	11%	9%	9%	5%	7%	15%	14%	13%	12%	11%	11%	31%		14%
304	19%	20%	19%	15%	14%	18%	8%	9%	10%	10%	11%	9%	9%	13%	13%	8%	11%	12%	16%	13%	16%	15%	13%	19%	14%	

Table 2J. Identities region 10 (without N-terminus region preceding first cysteine of the eight-cysteine pattern)

	407	155	201	383	23	255	159	160	163	164	162	161	185	175	209	272	71	276
407		99%	98%	32%	34%	35%	35%	35%	35%	35%	34%	35%	38%	38%	40%	37%	34%	33%
155	99%		99%	31%	33%	35%	35%	35%	35%	35%	34%	35%	38%	38%	40%	37%	34%	33%
201	98%	99%		32%	34%	35%	35%	35%	35%	35%	34%	35%	39%	39%	41%	37%	34%	33%
383	32%	31%	32%		78%	35%	35%	35%	34%	35%	33%	34%	34%	35%	37%	25%	26%	27%
23	34%	33%	34%	78%		37%	37%	37%	36%	36%	35%	36%	35%	35%	36%	27%	27%	25%
255	35%	35%	35%	35%	37%		99%	99%	99%	99%	96%	96%	53%	53%	45%	33%	33%	38%
159	35%	35%	35%	35%	37%	99%		98%	98%	98%	95%	95%	52%	52%	44%	33%	33%	38%
160	35%	35%	35%	35%	37%	99%	98%		98%	98%	95%	95%	52%	52%	45%	33%	33%	38%
163	35%	35%	35%	34%	36%	99%	98%	98%		98%	95%	95%	53%	53%	45%	33%	33%	38%
164	35%	35%	35%	35%	36%	99%	98%	98%	98%		95%	95%	52%	52%	46%	34%	34%	39%
162	34%	34%	34%	33%	35%	96%	95%	95%	95%	95%		91%	51%	51%	44%	33%	33%	39%
161	35%	35%	35%	34%	36%	96%	95%	95%	95%	95%	91%		53%	53%	46%	33%	33%	38%
185	38%	38%	39%	34%	35%	53%	52%	52%	53%	52%	51%	53%		99%	66%	34%	34%	34%
175	38%	38%	39%	35%	35%	53%	52%	52%	53%	52%	51%	53%	99%		66%	34%	34%	34%
209	40%	40%	41%	37%	36%	45%	44%	45%	45%	46%	44%	46%	66%	66%		33%	33%	34%
272	37%	37%	37%	25%	27%	33%	33%	33%	33%	34%	33%	33%	34%	34%	33%		91%	76%
71	34%	34%	34%	26%	27%	33%	33%	33%	33%	34%	33%	33%	34%	34%	33%	91%		76%
276	33%	33%	33%	27%	25%	38%	38%	38%	38%	39%	39%	38%	34%	34%	34%	76%	76%	

T

Table 2K. Identities region 11 (without N-terminus region preceding first cysteine of the eight-cysteine pattern)

	241	240	379	266	343	361	230	213	217	191	194	180	422
241		82%	41%	42%	44%	48%	44%	41%	39%	37%	40%	43%	47%
240	82%		44%	42%	43%	50%	48%	46%	42%	39%	40%	51%	49%
379	41%	44%		96%	50%	52%	54%	46%	44%	48%	44%	49%	47%
266	42%	42%	96%		50%	50%	51%	45%	45%	46%	43%	50%	47%
343	44%	43%	50%	50%		52%	54%	42%	39%	44%	39%	45%	50%
361	48%	50%	52%	50%	52%		87%	50%	48%	49%	49%	49%	52%
230	44%	48%	54%	51%	54%	87%		54%	46%	50%	49%	51%	55%
213	41%	46%	46%	45%	42%	50%	54%		46%	43%	38%	49%	46%
217	39%	42%	44%	45%	39%	48%	46%	46%		55%	56%	54%	55%
191	37%	39%	48%	46%	44%	49%	50%	43%	55%		51%	45%	46%
194	40%	40%	44%	43%	39%	49%	49%	38%	56%	51%		52%	51%
180	43%	51%	49%	50%	45%	49%	51%	49%	54%	45%	52%		56%
422	47%	49%	47%	47%	50%	52%	55%	46%	55%	46%	51%	56%	

Table 2L. Identities region 12 (without N-terminus region preceding first cysteine of the eight-cysteine pattern)

	26	19	385	25	265	20	346	323	189 (RodA)	248	195	216	176
26		99%	97%	87%	90%	89%	90%	79%	81%	82%	79%	82%	75%
19	99%		98%	86%	90%	90%	91%	80%	82%	83%	80%	83%	76%
385	97%	98%		88%	89%	88%	90%	80%	82%	83%	80%	83%	75%
25	87%	86%	88%		81%	80%	80%	74%	76%	77%	77%	78%	72%
265	90%	90%	89%	81%		99%	90%	80%	82%	84%	82%	80%	79%
20	89%	90%	88%	80%	99%		91%	80%	82%	84%	82%	80%	80%
346	90%	91%	90%	80%	90%	91%		82%	84%	86%	83%	82%	79%
323	79%	80%	80%	74%	80%	80%	82%		98%	96%	92%	86%	74%
189 (RodA)	81%	82%	82%	76%	82%	82%	84%	98%		98%	94%	88%	76%
248	82%	83%	83%	77%	84%	84%	86%	96%	98%		96%	89%	77%
195	79%	80%	80%	77%	82%	82%	83%	92%	94%	96%		87%	76%
216	82%	83%	83%	78%	80%	80%	82%	86%	88%	89%	87%		72%
176	75%	76%	75%	72%	79%	80%	79%	74%	76%	77%	76%	72%	
